# Action research at the BBC: Interrogating artificial intelligence with journalists to generate actionable insights for the newsroom

**DOI:** 10.1177/14648849251317150

**Published:** 2025-01-29

**Authors:** Bronwyn Jones, Rhianne Jones

**Affiliations:** University of Edinburgh, Edinburgh, UK; BBC Research and Development (R&D), Salford, UK

**Keywords:** Action research, participation, artificial intelligence, innovation, public service media, BBC

## Abstract

In this paper, we provide reflections from an embedded action research project undertaken at the UK’s largest public service broadcaster, the BBC, over a three-year period. It was aimed at eliciting research insights about the role and understanding of AI in news production and also intervening to engender change in the newsroom. We surface the messy realities of conducting this work, including the challenges to funding such long-term and resource-intensive research and the difficulties of measuring impacts. We include practical guidance to demystify the process of action-oriented research that strives for targeted change in news contexts, highlighting the need for researchers to cost in time for translational work and the importance of having ‘critical friends’ to hold them to account. Ultimately, we emphasise the value of action research in journalism studies and particularly at the nexus of news and technology. We argue for approaches that retain a critical perspective whilst closing the gap between theory, critique, and practice.

## Introduction

Technology is an increasingly important area for collaboration between scholars and news organisations. Journalists, like most practitioners, spend the vast majority of their time performing the tasks and requirements associated with their job and have limited dedicated time and resource for stepping out of these day-to-day pressures to reflect on the shifting terrain in which they work (Grubenmann, 2016). Particularly in daily news, the combination of a deadline-driven and high-paced environment with competitive and often precarious employment contexts can make it difficult to engage with technologies beyond instrumentalist training and practical use. But journalists are at the coalface of increasingly rapid technological change in media and communication technology that has seen successive advances in data-driven, algorithmic, and more recently artificial intelligence (AI) systems incrementally impact not only news production and distribution processes but also the wider information ecosystem in which journalism operates (Diakopoulos, 2019). Generative AI such as OpenAI’s series of GPT (text), DALL-E (image), Whisper (audio) and Sora (video) models are just the most recent in a long line of disruptive technologies. Journalists have for some time been grappling with deepfakes, synthetic media, and mis- and dis-information at scale, while incorporating into their working practices the (partial) automation of many daily activities like transcription, media monitoring, and data analysis, enabled by advances in machine learning (ML) and natural language processing (NLP). News organisations thus face a distinct tripartite challenge. Like other organisations, they must work out which of this new array of AI technologies (if any) is relevant and useful for them and how to procure or develop such systems in ways that align to their professional ethics and standards. Like others in the creative and knowledge industries, they must also work out how to deal with the proliferation of AI-generated material across the information ecosystem and grapple with the deployment of AI-driven systems by others in their supply chain. But unlike others, they also have the responsibility of reporting on the impacts and implications of AI for our societies.

In this complex context of pressing demands, connecting up productively with academics and their work across disciplines in relation to AI and emerging technologies is particularly important. For news workers and managers, this presents opportunities to access quality evidence-based analysis and argument but also to step outside of the logics and mindsets that prevail in working environments to consider different viewpoints and critiques. It can also help inform, upskill and empower editorial workers in an area in which they often feel underconfident, less than knowledgeable, and impotent to exert influence. Meanwhile, collaborations and other forms of bi-directional engagement around technology can benefit academia, informing the development of up-to-date curricula and maintaining networks of benefit to students in a fast-changing arena, providing access to data and contexts in which to test theses and better understand realities on the ground, and becoming routes for research to have demonstrable impact. However, this closer relationship can present risks and challenges for both parties, which can make them wary: for instance, of corporate influence or capture of research on the one hand, and unwanted exposure of internal and confidential activities on the other. Tensions are particularly heightened in sensitive and experimental areas such as AI, where reputational and commercial risks are high and ethical and professional norms are still being worked out.

In this paper, we provide reflections from an embedded action research project undertaken at the UK’s largest public service broadcaster, the BBC, over a three-year period. It was aimed at both eliciting research insights about the role and understanding of AI in news production, and also intervening to engender change in the newsroom. We want to surface the messy realities of conducting this work and focus on what we hope are helpful reflections about process and practice, whilst offering a little insight into what we have learnt about AI in public service media journalism from this approach. We provide practical guidance that aims to demystify the process of engaged and action-oriented research that strives for targeted impact in news contexts. We argue for action research approaches that retain a critical perspective whilst closing the gap between theory, critique, and practice.

## Context and challenge

Uncritical experimentation with AI in newsrooms risks replicating and exacerbating some of the problematic tendencies seen in news organisations’ long history of ‘innovation’ (Prenger and Deuze, 2017). For instance, lacking long-term strategic thinking and failing to meet community needs (Lewis et al., 2024), being mobilised to capture the democratic aspirations of digital journalism in favour of market-oriented solutions (Creech and Nadler, 2017) and focusing attention on the novel over maintenance of important things people value (Vinsel and Russell, 2020). Artificial intelligence is a catch-all term given to a wide range of data intensive computational systems that have been increasingly commercialised and deployed across sectors, including by newsrooms around the world (Beckett and Yaseen, 2023; Broussard et al., 2019) and in public service media (EBU, 2023). These systems involve more than the word technology suggests - they entail “a technical and social practice, institutions and infrastructures, politics and culture” (Crawford, 2021: 8). Generative AI is a branch of AI that describes systems that can produce “new” content (text, imagery, audio, data) based on user inputs known as prompts and the datasets they have been trained on. The complex, opaque, and inscrutable nature of many AI systems makes it difficult for journalists to make sense of them, scrutinise them, and ensure they are using them responsibly in ways that support editorial values (Jones et al., 2022). The unrealistic and often fantastical narratives of AI that dominate popular imagination (Cave and Dihal, 2019; Elish and boyd, 2017) obfuscate the human and material infrastructures that support these systems (Crawford, 2021) and the real harms they can cause to people and planet (Acemoglu, 2021; Dhar, 2020). Additionally, the growing dependency of newsrooms on big tech platforms and more recently on providers of AI tools and infrastructures complicates the task of ensuring new technologies work in the interests of news organisations and the publics they serve (Simon, 2023). These dynamics, coupled with the recent prevalence of polarised and sensationalised narratives of hype and doomerism (Roe and Perkins, 2023) also make it difficult to report on AI for non-specialists, even as the need to interrogate the myriad ways AI systems are impacting publics - for good and ill – grows (Narayanan and Kapoor, 2024).

For news organisations, two core and interconnected challenges emerge in this context: 1) understanding AI and 2) directing socio-technical change in relation to AI. Responding to these challenges requires bridging academic knowledge and newsroom practice, as well as conducting multidisciplinary research and cross-team working in a timely way. Notable progress has been made in this arena since our own work began in 2020, with multistakeholder groups developing practical guides and web-hosted toolkits for newsrooms (e.g. Partnership on AI, 2023, 2024), guidelines developed by and for newsrooms (Becker et al., 2023; RSF, 2023), and academic engagement aimed at stimulating interventions (e.g. around literacy from Deuze and Beckett, 2022; Jones et al., 2022). But significant barriers remain to improving this dialogue, including misaligned incentives and timeframes (e.g. scholarly publication over months and years vs actionable insights over days and weeks), epistemological tensions (e.g. theoretical knowledge coming up against experiential knowledge), and silos (of academic departments as well as organisational teams). These barriers can be compounded by, on the one hand, practitioner critiques that journalism scholarship is detached from the needs of the news industry and exploitative of those being studied, and on the other hand, researcher reticence to be co-opted into serving industry priorities and commercial search for profit, or compromising academic independence (Bélair-Gagnon and Usher, 2021). Openness to academic engagement and perception of the value of academic work varies across country, organisation, and even across internal team/unit (see Nyre and Maiden, 2022, for an example of such challenges in a cross-country EU innovation project). Comparison is however difficult beyond anecdotal accounts, for instance of journalists weary of academics in the US (Bélair-Gagnon and Usher, 2021) and engaged with the academy in for example, the Nordic countries.

Our work represents an attempt to respond to some of these challenges and barriers in a UK-specific context, which we describe below.

## Case study context

Our qualitative study was conducted in the UK’s largest public service broadcaster, the BBC, over a period of three years from 2020-2023. It was funded as three projects, the first focusing on research and the following two as impact projects focused on collaboration, co-design and intervention. In 2023, the BBC was the most widely used source of news in the UK both online and offline, and one of the most highly trusted sources of news (Nielsen et al., 2023). It is a unique site for study, which stands apart from the wider commercial news media sector due to its public service commitments and license fee-funding model, but also from other public service media due to its size and national and global reach (unlike peer organisations, in the US for example), and its historic role in setting technical and editorial standards for the media sector. Its public service ethos involves commitment to values such as due impartiality, universality, and public accountability and extends to a commitment to innovate technologically in the public interest (BBC R&D, n.d.). Unlike commercial news media, the BBC has relatively stable funding through a licence fee, replacing the necessity to make profit for shareholders with the requirement to demonstrate value for money and broad engagement across society, amongst other commitments including impartiality and diversity (Royal Charter 2016, Cm 9365).

Driven by public service values and goals, this context has engendered a unique character to its culture of innovation and shaped many of its contributions to technology development over the century since its incorporation (e.g. computing education from the Micro computer to Microbit, infrastructure development for digital transition, advancing media accessibility for those with disabilities). This does not mean it entirely escapes the tendency to mimetic isomorphism we see across the news industry in current and earlier waves of innovation (e.g. around AI and AI policies see Simon, 2024 and Becker et al., 2023; video see Kalogeropoulos and Nielsen, 2018; metrics see Christin, 2020). For instance, goals of reach and engagement designed in the BBC’s case to meet commitments to universality, diversity, and value have often led to deployment of the same techniques and approaches to attract and retain audiences as the wider industry, with use of similar metrics and measures of success. However, it does mean the organisation is predisposed to innovating in the public interest rather than for financial gain, to advancing knowledge, and to collaborating with partners such as academics with shared civic goals. This makes it generally a welcoming environment for researchers, with a number of formalised structures for managing partnerships and collaborations in place to aid the process and to ensure academic independence is balanced with providing value for the organisation. However, its national significance and market role means it is also oversubscribed as a site of study and its public service position intensifies scrutiny, so awareness of the potential for reputational damage can also make the organisation risk averse in this area. Our experiences will of course be coloured by this distinctive context. For instance, working with R&D practitioners who hold shared ideas about the value of research smooths the process and provides a cohort eager for insights. This is less the case with newsworkers who in our experience generally range from sceptical of the practical utility of research to mildly interested but not invested, with a select few (either familiar with research practices or with a clear stake in the topic) highly motivated to engage.

Our research period of 2020-2023 was characterised by an increasing recognition of the growing relevance of AI for the BBC, greater internal application of machine learning, and maturing of governance of AI. By 2024, this had led to the BBC publishing its first editorial guidance on the use of AI (BBC, n.d.) and set of AI Principles (BBC, 2024) and launching mandatory AI training for staff.

## Positionality of authors

The dual role of the two researchers on this project is an important factor shaping the work and access to the research environment. We are both academic researchers but also employees of the BBC; at the time of writing one was a researcher in BBC R&D and formerly 10 years a journalist for BBC News, whilst the other was Research Director of the Responsible Innovation Centre at BBC R&D. As such, we have ‘skin in the game’, which has implications of significance for our work and for how others understand it. There are potential risks and disadvantages to this arrangement, such as being unknowingly biased or too familiar and bound by custom to elicit tacit knowledge (Holmes, 2020). However, the benefits are that it brings independent academic research into conversation with engaged practice and embedded expert knowledge. The authors had the advantages of easy access to research contexts, a priori knowledge, trust from participants, and claims to producing authentic or ‘thick’ description (Geertz, 1973). We strived to combine the researcher-analyst and the practitioner-informant viewpoints, spanning etic and emic knowledge (Haapanen and Manninen, 2023), and question the dichotomous framing of the researcher as either insider or outsider. As reflexive methodologies in anthropology, sociology and action research have long advocated, it behoves us to be reflective about the implications this has for the topics and methods we choose and conclusions we draw (Hersted et al., 2021). We recognise that our affiliation conferred a level of credibility with practitioners that is unlikely to be readily available to many other researchers and that this may mean our recommended best practices may not hold in their circumstances. We try to unpack some of this in the following sections.

## The research and intervention process

We identified sites of study and mechanisms for intervention through engagement with theory and prior academic work, combined with our own empirical research and elements of co-production with journalists, and insights from our professional experiences. First, we sought an informed understanding of the context. We combined 1) desktop research and document analysis to explore use of AI in the BBC and wider news industry, with 2) semi-structured open-ended interviews with 14 journalists about their understandings of, and attitudes toward AI (Jones et al., 2022), and 3) a workshop bringing industry and academic contributors into structured discussion with one another about the role and implications of AI in journalism. This was supplemented by informal discussion with internal teams working in news and technology groups and field notes. From this we identified the intelligibility of AI as a core challenge which compromised newsworkers’ agency. We identified 1) a need for news workers to be more involved in AI research and development processes as a way to ensure editorial, ethical, and professional requirements and cultures are meaningfully integrated and public interest goals and values are centred in the innovation process, and 2) the concomitant need for newsworkers to develop a critical AI literacy as a foundation for contributing to AI development, for responsible news production practice using AI, and for effective news reporting of AI. In response, we devised approaches to build their capacity for understanding and leveraging their professional expertise in relation to AI, using co-design methods that considered plausible futures and the ethical and professional questions they raised. We also co-created recommendations for an AI engagement strategy at the organisation.

At that point, in November 2022 generative AI was becoming a topic of interest and concern for the organisation and its journalists following the release of Open AI’s ChatGPT. In response, we hosted a multi-stakeholder workshop with news managers, journalists, technologists and academics to scope the new challenges this raised and combined these insights with desktop research to write a rapid review of the risks generative AI posed for journalism to feed back to participants and the wider community as a resource (Jones et al., 2023). We then hosted three speculative co-design workshops, each involving between 6-10 BBC journalists in which we explored the ethical and professional implications of present and future applications of AI in news production. This was inspired by futures thinking methods from design and human computer interaction research (e.g. Harrington and Dillahunt, 2021) and capacity building methods from education and literacy studies which mobilise community members’ skills and knowledge. We co-created visual artefacts with journalists and technologists, then used them to inform and engage other practitioners about AI. This included materials like a toolkit for doing futures thinking with journalists (Jones and Jones, 2023), an infographic, animated video, and storyboards. Finally, we ran two interactive public engagement sessions to share some of what we had learnt and hear the questions and concerns raised by members of the public – the people who would ultimately be impacted by AI in journalism.

## Theory and method: Soft infrastructures, action research and participation

Recognising the tendency for journalism innovation, and some studies of technology and innovation in journalism, to focus on the latest shiny new thing over longer-term and more strategic questions (Hermida and Young, 2021), we mobilised the concept of infrastructure from science and technology studies (STS) to inform our choice of sites and subjects of research. Journalism studies has long been engaging with STS to apply sociotechnical approaches to better conceptualise the interrelationships between human actors and nonhuman technological actants (see e.g. engagement with actor-network theory in Schmitz Weiss and Domingo, 2010 or Lewis and Westlund’s development of the ‘Four A’s’ framework in 2015). Simply, infrastructure refers to a structure underlying or supporting something - the physical and organisational elements that operate as a system on which other things operate. This lens can help identify what enables a basis of stability on which AI-related innovation activity builds and home in on what is continual alongside what is contingent. Defining what constitutes infrastructure worth studying is a categorising moment which delineates which parts of heterogeneous and overlapping networks to draw out for analysis (Larkin, 2013). A lot of news organisations’ efforts, and many studies of AI in news production, have focused on ‘hard infrastructure’, including the material elements like devices, software, staff, data sets, licenses/contracts etc. required to make AI work in practice. Less well understood is the ‘soft infrastructure’ including social and psychological resources such as relationships and inter-personal networks, tacit information and knowledge, organisational culture and training (Bowker and Star, 1999). This is where we focused our research, exploring the attitudes and expectations of AI amongst journalists, as well as communally available resources.

Action research has been a fruitful approach in studies of journalism innovation and as Grubenmann explains, it “offers a framework for research collaborations between scholars and practitioners, generating holistic and solution-oriented outcomes of value for science and practice” (2016: 160). Wagemans and Witschge (2019) argue that by allowing researchers to experience the phenomena they study, it enables them to do justice to the complexity of the media landscape and be flexible in dynamic contexts. We started from an action research orientation that brings together action and reflection, theory and practice in the pursuit of practical solutions to issues of pressing concern (Bradbury, 2015). Within this, we foregrounded forms of participation that aim at researching ‘with’ rather than ‘on/about’ newsworkers. Our approach was iterative and pragmatic, aiming to respond to the needs, expectations and insights from the context while balancing external constraints and enablers such as available funding and resource. This required close collaboration to garner the meaningful participation of news teams. We believe this approach can engender the self-referential awareness and “shared storytelling and world-making” that elicits more of the rich diversity of experiences of journalism (even within well-studied legacy organisations like the BBC) and recognises our complicity as researchers in journalism’s future (Witschge and Deuze, 2020).

Participatory approaches to AI with both professionals and publics are gaining traction as they can help centre values of inclusion and plurality, and develop community empowerment, notably among marginalised groups (Birhane et al., 2022; Park, 2022). Journalists are now often expected to integrate into their workflows AI systems that have not been designed with the values, standards, needs or expectations of journalism in mind. Our focus was on bringing editorial workers into the collective conversation about AI in their organisation as a response to the observation that their voice(s) and professional knowledge and expertise have been surprisingly absent or deprioritised in discussion of AI development and application, which is dominated by technical priorities. Internal industry engagement is often aimed at either eliciting technical requirements from them for developers (for example to improve performance or efficiency of an AI system) or training them to be proficient in using an AI tool or system. In contrast, we wanted to identify what editorial workers’ perspectives on AI were and what challenges they faced in order to respond in ways that help and empower them. It is important to note that though our focus was on newsworkers, we recognised that addressing AI in journalism requires multidisciplinarity and strove to bring different expertise into conversation with each other, including technical, legal, policy, managerial, design, academic, and editorial.

## Reflections on challenges

Of the many challenges we faced, we draw out three to focus on: the first is the core problem we identified for journalists on the ground, the second two are challenges facing scholars conducting and evaluating action research.

### Making AI visible

Currently most journalists engage with AI systems as ‘users’ of pre-made tools and end up as ‘observers’ of AI in the news industry, rather than as contributors to directing the future shape of their profession or practitioners with agency in determining how newsroom technology is developed, negotiated or in fact resisted. A fundamental barrier to their understanding of AI which can in turn lead to disengagement, is how invisible and abstract AI seems to them. Work in the field of explainable AI (XAI) has taken on the important task of interpreting and articulating the decision-making and outputs of black-box models (Mittelstadt, 2021), which it is hoped will contribute to the ability of journalists and their organisations to make sense of AI systems’ predictions, classifications and decisions (e.g. Simkute et al., 2021). Such work is complicated by the complexity of those systems, with some scholars noting that even system developers of such systems have difficulty in explaining why their systems produce specific outputs. This holds true for generative AI, which can produce very unpredictable outputs. Nevertheless, we identified a more foundational gap for many journalists of identifying where AI was already present in their work environment and where it might be in future, what that meant for them and their profession, why it mattered, and what they might do about it.

### Building bridges to make collaboration possible

The metaphor of a bridge - used in the ‘Bridging the Impact’ title of the special issue of which this article is part - is particularly salient. Like building a bridge, action research is labour- and resource-intensive, it requires joint effort from both sides – building from each direction to meet in the middle, it provides a stable route for the bi-directional exchange of ideas and information, but also requires maintenance to remain stable and is strained when there is misalignment. Keeping partners interested in competition with their day-to-day pressures and incentives is a constant challenge, as is ensuring researchers are sufficiently “empathic and involved in the perspectives of the problem-owners” (Nyre and Maiden, 2022). Both academia and journalism are “rife with inscrutable rules and customs”, including those related to professional ethics that can make collaboration ripe for misunderstanding (Davis, 2021). In our case, the level of access and resource required to enable meaningful participatory action research was significant and required both parties to commit to ongoing support over time. The messy reality of funding this work involved continually bidding for further funding to specifically target knowledge exchange and impact activities to enable us to further pursue our interventions, as these goals fell outside of the boundaries of standard research activity and the initial research grant. It also required dedicated time allocated from the BBC side as in-kind support to provide the labour of workers whose time and intellectual effort was core to its success. Where possible, we also provided recompense in the form of vouchers.

This bi-directional exchange of ideas and information also necessitated what we refer to as translational work: communicating to diverse groups using language and repertoire familiar to them in order to convey unfamiliar concepts or ways of thinking necessary to understand each other’s worlds. This work, for instance between technical and editorial staff, legal and product experts, academics and industry practitioners, took a particularly large amount of time and dedication as a pre-requisite for building and maintaining the relationships needed. It involved listening and learning by the researchers, facilitating others to share perspectives, and finding apposite moments to feed back critique without mis-representing or offending participants. However time-consuming, this was viewed as a benefit within the organisation, since the research was not directed along organisational lines or siloed into existing structures, so it brought an external view on an institutional challenge but mobilised knowledge from internal teams to identify actions that could be taken in response – from upskilling newsworkers to making recommendations for future editorial guidance.

### Measuring impact: A bridge too far?

The biggest challenge we faced was measuring the impacts of interventions and engagements, whether intended or unintended, desirable or problematic. Sustained engagement with individual journalists or specific cohorts was not possible due to constraints on their time so we could not gauge how their understanding and actions changed over time or gather sufficient in-depth feedback. When we tried to gauge whether changes in understanding or perspective had occurred by using lightweight online surveys before and after interventions, we received very limited responses from which we could not reliably draw conclusions, though a number of participants pro-actively contacted us with thanks and further thoughts. Our action research approach was a complicating factor here, and this problem has been found by others across sectors (e.g. healthcare, Cook et al., 2017). We believe the tactical nature of responding to live developments helped us achieve impact in practice and we received informal recognition of how our interventions shaped thinking in the organisation, but it made applying formal evaluation more difficult in practice. This limited our ability to evidence the utility of our approach both academically and to industry, which often favour structured and quantitative data in their reporting over the more narrative approach we adopted. This could potentially be an even greater barrier for researchers without the insider position from which we benefited, if journalists felt less collegial obligation to give feedback or less investment in the work due to a greater perceived distance between themselves and the researchers.

## Recommendations and best practices

Here we provide recommendations for successful action research based on our experiences: the first pertains specifically to exploring AI in newsrooms, the rest apply to action research in journalism more broadly.

### Create tangible resources to ‘apprehend’ AI

We found design methods, including participatory and speculative approaches, to be useful for addressing the problem of how to make AI more real, visible, and comprehensible to non-experts due to the field’s focus on contextualising AI systems within the environments which make them meaningful for people. We trialled co-design activities to help journalists visualise not only the material and immaterial components that constitute AI but make clear their relevance by mapping them to editorial jobs and tasks and linking to editorial standards, values and goals. By drawing directly from journalists’ own questions, concerns, stories, and use of language, we could develop informative and context-sensitive resources to build understanding and critical faculties with other journalists in a way that resonated. This included a re-usable toolkit including visualisations of familiar tools with unfamiliar capabilities, such as an AI-augmented content management system, and storyboards for facilitated discussion around current case studies and potential future impacts (Jones and Jones, 2023, see Supplemental Material). Crucially, we found that this builds capacity for discernment – the ability to judge things clearly – and for critique. It enabled us to identify priorities from the ground up, such as the need for care and maintenance of core systems, conventions, values and standards - the things journalists wanted to keep and improve and not 'innovate away’ either deliberately or accidentally. For example, workshops on generative AI highlighted divergences between nuanced notions of accuracy, bias, (due) impartiality, and independence as understood and applied in editorial terms, versus their application and operationalisation in AI development and deployment. Through discussion of these differences, journalists were able to anticipate points of misalignment between accepted professional practice and the operations of a particular AI system. Other methods in this toolkit include scenario writing and participatory foresight (Kieslich et al., 2024), design ethnography (Gutierrez Lopez et al., 2022) and design thinking with co-creation (Portugal et al., 2023).

Our aim was not to manufacture legitimacy for AI in the newsroom nor to disavow its potential utility but to develop critical capacity amongst editorial workers to develop informed perspectives and be able to bring their professional expertise to bear on discussions about AI and their engagement and interactions with AI systems in their daily work. We view this as important in the public service media context of the BBC, as it could contribute to resisting the tendency for mimetic isomorphism - imitating the actions of other actors in the industry (Karlsson et al., 2023), instead empowering newsworkers to consider alternative ways of working with AI that better support their public interest goals.

### Develop trusted collaborations through mutual respect

Journalism scholars are positioned to critique not out of hostility but out of a longing to see improvement by using research to challenge journalists to improve their practices in particular and concrete ways (Carlson, 2021). As Carlson explains, this engagement “at once encompasses accord and conflict, promise and action” (Ibid: 236). For action researchers, this involves a balancing act of building trust while retaining space for critique that may be hard to hear. This relies on developing mutual respect, which in turn relies on a degree of understanding of each other’s motivations and identification of shared goals/values. In our case, we emphasised the shared goal of improving public interest journalism for both publics and practitioners in the face of volatility related to AI development and associated automation. Establishing an agreed grounding in constructive critique is essential, whereby collaborative partners and participants feel equally as empowered to question and challenge the researcher(s).

### Factor in time and resource for translational work and be willing to iterate

Change-focused work requires time and resource to be factored into research plans in order to turn research findings into actionable insights, and to build, maintain and mobilise relationships that support researchers to translate the significance and value of the research into culturally appropriate forms for the context. Moreover, action research requires translational activities to be woven into the project, rather than simply ‘tagged on’ at the end, as is the more common approach to impact-focused activity. Our approach to dealing with this challenge was to combine multiple complementary streams of funding but we recognise that this was difficult, time-consuming and messy – with rejections, revisions and delays impacting our ability to retain momentum. Funders (or for student researchers, their supervisors) may need to be convinced of the necessity of significant allocation of time and money to translational activities, which requires clear and strong articulation of their benefit and even then, may not be funded. As Wagemans and Witschge explain, innovation processes are non-linear, iterative and converged and “rather than assuming there is a fixed place that we can research, we need to attempt to move with the actual objects and subjects of study as much as we can” (2019: 213). Though this can make the process sound very contingent and uncertain, over time, as the work became established, this conceptual ‘bridge’ became a form of infrastructure, in that it became invisible and accepted. For instance, our engagements led to us being proactively invited to feed into discussions around AI strategy, brief teams, conduct educational activities, and contribute to technical and multidisciplinary communities of practice. Often, by the time a news organisation knows it needs to address a question, it is already a near-term problem, which leaves little time for researchers scoping, planning, collecting, analysing data – and then doing the translational work necessary to maximise the chance of influencing at the opportune moment. The iterative approach inherent to action research helped us identify questions coming up ahead for the organisation which it was not yet thinking about and help prepare them to act. We do recognise these routes may well have been more open to us due to our roles within the industry partner, but believe there remains a lesson to be learnt here about openness to adapting to the cadence of the research environment and being willing to apply research insights in unforeseen areas (e.g. policy, professional training etc.).

### Balance planning and flexibility in impact assessment

We recommend a balance of planning and flexibility in assessing impact and change, with a prepared selection of routes and methods for gathering feedback devised (e.g. during interviews and workshops, in conversation and recorded via field notes, testimonials by email and survey) in advance to be deployed flexibly and combined where appropriate to get to the heart of what influence the work has had in both the short and long-term. We believe that varying techniques to accommodate participant preferences also minimises the load on them and helps the process feel less extractive. Forms of participatory research have been critiqued for the illusion of consultation and reproduction of existing dynamics rather than challenging or reconfiguring them (Cahill, 2007). We recognise that participatory approaches also pose the risk of extractive, instrumentalist and exploitative engagement that fails to give back to communities, change the status quo or redistribute power (Birhane et al., 2022; Sloane et al., 2022). We recommend ensuring participants understand the reasons for gathering data about impact and where appropriate, amplifying their voices (of praise and critique) so lessons can be learnt and they can see the manifest value in their involvement.

### Create a role for critical friends

Protocols for critique are particularly pertinent for journalism action research and other highly collaborative projects. We recommend putting in place independent advisors or ‘critical friends’ with whom questions, concerns and reflections can be shared and discussed as a way to ensure researchers have a place for dialogue with peers who are not involved in the project and benefit from their distance and ability to hold them to account (Baskerville and Goldblatt, 2009; Hersted et al., 2021). In our case, we felt it was incumbent on us as researchers working at the institution we were studying, to continually consider the motivations underpinning our choices and potential conflicts of interest, perceived or otherwise, that may occur. We relied on informal relationships with academic colleagues to discuss such issues but this was a form of largely unrecognised voluntary labour from them and did not allow for an optimal extent or regularity of discussion. We suggest formally organising a network of critical friends and scheduling regular check-ins for reflection throughout the project.

Our recommendations inevitably reflect our own experiences and we recognise they may not translate as effectively into the work of other researchers who may have a more distant or short-term relationship with the organisation being studied and who therefore may be perceived more strongly as outsiders and treated differently.

## Conclusion: An agenda for research and change

Making select applications of AI useful and appropriate for journalism and making public interest journalism resilient in an age of proliferating AI, will require incremental and thoughtful change led by multidisciplinary teams with journalists and editorial experts front and centre. Independent and critical academic research will be a crucial voice in this arena that can provide a countervailing force to hype and technological determinism. But effective mobilisation of scholarly research that does more than study and critique from afar is also needed. Impact-oriented research in applied disciplines like journalism has a long history but grappling with AI and other emerging technologies will require robust academic-industry collaborations that span disciplines. Action research approaches that retain a critical perspective whilst closing the gap between theory, critique, and practice have much to offer in the study of all emerging technologies in journalism. For this to flourish, researchers must make explicit the normative underpinnings and the practical messiness of their work in order to bring them into a shared space for discussion and scrutiny. Like the earliest of bridges which were just stepping stones, we hope with this work we have charted pathways on which others can build.

## Supplemental Material

Supplemental Material - Action research at the BBC: Interrogating artificial intelligence with journalists to generate actionable insights for the newsroom Supplemental Material for Action research at the BBC: Interrogating artificial intelligence with journalists to generate actionable insights for the newsroom by Bronwyn Jones and Rhianne Jones in Journalism

## References

[bibr1-14648849251317150] Acemoglu D (2021) Harms of AI. *Working Paper Series* . DOI: 10.3386/w29247.

[bibr3-14648849251317150] Baskerville D Goldblatt H (2009) Learning to be a critical friend: from professional indifference through challenge to unguarded conversations. Cambridge Journal of Education 39(2): 205–221.

[bibr4-14648849251317150] BBC (2024) BBC AI Principles. https://www.bbc.co.uk/supplying/working-with-us/ai-principles/.

[bibr5-14648849251317150] BBC (n.d.) Guidance: the use of artificial intelligence. Retrieved 02.29.2024 from. https://www.bbc.co.uk/editorialguidelines/guidance/use-of-artificial-intelligence.

[bibr6-14648849251317150] BBC R&D (n.d.) Our purpose. Retrieved 02.15.2024 fro. https://www.bbc.co.uk/rd/about/our-purpose.

[bibr7-14648849251317150] Becker KB Simon FM Crum C (2023) Policies in parallel? A comparative study of journalistic AI policies in 52 global news organisations. Pre-print ahead of peer review DOI: 10.31235/osf.io/c4af9.

[bibr8-14648849251317150] Beckett C Yaseen M (2023) Generating Change: A Global Survey of what News Organisations are Doing with AI. Report by JournalismAI. Retrieved from. https://www.journalismai.info/s/Generating-Change-_-The-Journalism-AI-report-_-English.pdf.

[bibr9-14648849251317150] Bélair-Gagnon V Usher N (2021) Introduction: improving journalism with academic research. In: Bélair-Gagnon V Usher N (eds) Journalism Research that Matters. online edn. New York: Oxford Academic. DOI: 10.1093/oso/9780197538470.003.0001.

[bibr10-14648849251317150] Birhane A Isaac W Prabhakaran V , et al. (2022) Power to the People? Opportunities and Challenges for Participatory AI. EAAMO ‘22. New York, NY: Association for Computing Machinery, 1–8. DOI: 10.1145/3551624.3555290.

[bibr63-14648849251317150] Bowker GC Star SL (1999) Sorting Things Out: Classification and Its Consequences. The MIT Press.

[bibr11-14648849251317150] Bradbury H (ed) (2015) The Sage Handbook of Action Research. London: Sage.

[bibr12-14648849251317150] Broussard M Diakopoulos N Guzman AL , et al. (2019) Artificial intelligence and journalism. Journalism & Mass Communication Quarterly 96(3): 673–695. DOI: 10.1177/1077699019859901.

[bibr64-14648849251317150] Cahill C (2007) Repositioning ethical commitments: Participatory action research as relational praxis of social change. ACME: An International Journal for Critical Geographies 6(3): 360–373. doi: 10.14288/acme.v6i3.784.

[bibr13-14648849251317150] Carlson M (2021) ‘Conclusion: betrothed or belligerent: what type of engagement do we need?’, in Valérie Bélair-Gagnon Nikki Usher (eds), Journalism Research that Matters (New York, 2021; online edn, Oxford Academic, 22 July 2021), DOI: 10.1093/oso/9780197538470.003.0019.

[bibr60-14648849251317150] Cave S Dihal K (2019) Hopes and fears for intelligent machines in fiction and reality. Nature Machine Intelligence 1: 74–78. doi: 10.1038/s42256-019-0020-9.

[bibr14-14648849251317150] Christin A (2020) Metrics at Work: Journalism and the Contested Meaning of Algorithms. Princeton: Princeton University Press.

[bibr15-14648849251317150] Cook T Boote J Buckley N , et al. (2017) Accessing participatory research impact and legacy: developing the evidence base for participatory approaches in health research,. Educational Action Research 25(4): 473–488. DOI: 10.1080/09650792.2017.1326964.

[bibr16-14648849251317150] Crawford K (2021) Atlas of AI: Power, Politics, and the Planetary Costs of Artificial Intelligence. London: Yale University Press.

[bibr17-14648849251317150] Creech B Nadler AM (2017) Post-industrial fog: reconsidering innovation in visions of journalism’s future. Journalism 19(2): 182–199.

[bibr18-14648849251317150] Davis C (2021) How academics can work with journalists (hint: they already have). In: Bélair-Gagnon V Usher N (eds) Journalism Research that Matters. Online edn. New York: Oxford Academic. DOI: 10.1093/oso/9780197538470.003.0016.

[bibr19-14648849251317150] Deuze M Beckett C (2022) Imagination, algorithms and news: developing AI literacy for journalism. Digital Journalism 10(10): 1913–1918. DOI: 10.1080/21670811.2022.2119152.

[bibr20-14648849251317150] Dhar P (2020) The carbon impact of artificial intelligence. Nature Machine Intelligence 2: 423–425.

[bibr21-14648849251317150] Diakopoulos N (2019) Automating the News: How Algorithms Are Rewriting the Media. Cambridge, MA: Harvard University Press.

[bibr22-14648849251317150] EBU (2023) Artificial intelligence - public service media leveraging AI. Members Only Report. Retrieved 02.29.2024 from. https://www.ebu.ch/files/live/sites/ebu/files/Publications/MIS/members_only/psm/EBU-MIS-ARTIFICIAL_INTELLIGENCE_AND_PSM.pdf.

[bibr62-14648849251317150] Elish MC boyd d (2017) Situating methods in the magic of Big Data and AI. Communications Monographs 85(1): 57–80. doi: 10.1080/03637751.2017.1375130.

[bibr23-14648849251317150] Geertz C (1973) The Interpretation of Cultures. Basic Books.

[bibr24-14648849251317150] Grubenmann S (2016) Action research. Digital Journalism 4(1): 160–176. DOI: 10.1080/21670811.2015.1093274.

[bibr25-14648849251317150] Gutierrez Lopez M Porlezza C Cooper G , et al. (2022) A question of design: strategies for embedding AI-driven tools into journalistic work routines. Digital Journalism 11(3): 484–503. DOI: 10.1080/21670811.2022.2043759.

[bibr26-14648849251317150] Haapanen L Manninen VJ (2023) Etic and emic data production methods in the study of journalistic work practices: a systematic literature review. Journalism 24(2): 418–435. DOI: 10.1177/14648849211016997.

[bibr27-14648849251317150] Harrington C Dillahunt TR (2021) Eliciting tech futures among black Young adults: a case study of remote speculative Co-design Proceedings of the 2021 CHI Conference on Human Factors in Computing Systems (CHI '21). New York, NY: Association for Computing Machinery, 1–15. Article 397 DOI: 10.1145/3411764.3445723.

[bibr61-14648849251317150] Hermida A Young ML (2021) Journalism Innovation in a Time of Survival. In: Luengo M Herrera-Damas S (eds). News Media Innovation Reconsidered: Ethics and Values in Creative Reconstruction of Journalism. Wiley.

[bibr28-14648849251317150] Hersted L Ness O Frimann S (eds) (2021) Action Research in a Relational Perspective: Dialogue, Reflexivity, Power and Ethics. Routledge.

[bibr29-14648849251317150] Holmes AGD (2020) Researcher positionality - a consideration of its influence and place in qualitative research. Shanlax International Journal of Education 8(4): 1–10.

[bibr30-14648849251317150] Jones B Jones R (2023) Futures thinking with journalists: resource pack for researchers and innovators in the news industry. Interactive Workbook. Available at: https://www.pure.ed.ac.uk/ws/portalfiles/portal/408001564/Interactive_Futures_Thinking_with_Journalists_Toolkit.pdf.

[bibr31-14648849251317150] Jones B Jones R Luger E (2022) AI ‘everywhere and nowhere’: addressing the AI intelligibility problem in public service journalism. Digital Journalism 10(10): 1731–1755. DOI: 10.1080/21670811.2022.2145328.

[bibr32-14648849251317150] Jones B Luger E Jones R (2023) Generative AI & journalism: a rapid risk-based review. Industry Report. Available at: https://www.pure.ed.ac.uk/ws/portalfiles/portal/372212564/GenAI_Journalism_Rapid_Risk_Review_June_2023_BJ_RJ_EL.pdf.

[bibr33-14648849251317150] Kalogeropoulos A Nielsen RK (2018) Investing in online video news. Journalism Studies 19(15): 2207–2224. DOI: 10.1080/1461670X.2017.1331709.

[bibr34-14648849251317150] Karlsson M Ferrer Conill R Örnebring H (2023) Recoding journalism: establishing normative dimensions for a twenty-first century news media. Journalism Studies 24(5): 553–572. DOI: 10.1080/1461670X.2022.2161929.

[bibr36-14648849251317150] Kieslich K Diakopoulos N Helberger N (2024) Anticipating impacts: using large-scale scenario-writing to explore diverse implications of generative AI in the news environment. AI Ethics. DOI: 10.1007/s43681-024-00497-4.

[bibr38-14648849251317150] Larkin B (2013) The politics and poetics of infrastructure. Ann Review Anthropol 42: 327–343.

[bibr39-14648849251317150] Lewis SC Westlund O (2015) Actors, actants, audiences, and activities in cross-media news work. Digital Journalism 3(1): 19–37. DOI: 10.1080/21670811.2014.927986.

[bibr40-14648849251317150] Lewis SC Hermida A Lorenzo S (2024) Jobs-to-Be-Done and journalism innovation: making news more responsive to community needs. Media and Communication 12: 7578. DOI: 10.17645/mac.7578.

[bibr41-14648849251317150] Mittelstadt B (2021) Interpretability and transparency in artificial intelligence. In Véliz C (ed.), The Oxford Handbook of Digital Ethics (Online edn, Oxford Academic, 10 Nov. 2021), DOI: 10.1093/oxfordhb/9780198857815.013.20, Available at SSRN: https://ssrn.com/abstract=4317938.

[bibr42-14648849251317150] Narayanan A Kapoor S (2024) AI Snake Oil: What Artificial Intelligence Can Do, what it Can’t, and How to Tell the Difference. Princeton University Press.

[bibr43-14648849251317150] Nielsen RK Schulz A Fletcher R , et al. (2023, 17 August) The BBC is under scrutiny. In: Here’s what Research Tells about its Role in the UK. Reuters Institute. https://reutersinstitute.politics.ox.ac.uk/news/bbc-under-scrutiny-heres-what-research-tells-about-its-role-uk.

[bibr44-14648849251317150] Nyre L Maiden N (2022) Can action research improve local journalism? A critical evaluation of the EU Innovation Action INJECT Norway. Nordicom Review 43(2): 171–189. DOI: 10.2478/nor-2022-0011.

[bibr45-14648849251317150] Park TM (2022) Making AI inclusive: four guiding Principles for ethical engagement. White Paper, Partnership on AI. https://partnershiponai.org/wp-content/uploads/dlm_uploads/2022/07/PAI_whitepaper_making-ai-inclusive.pdf.

[bibr46-14648849251317150] Partnership on AI (2023) AI adoption for newsrooms: a 10-step guide. https://partnershiponai.org/download/10059/?tmstv=1699987495.

[bibr47-14648849251317150] Partnership on AI (2024) Local news. https://partnershiponai.org/workstream/local-news/.

[bibr49-14648849251317150] Portugal R Wilczek B Eder M , et al. (2023) Design thinking for journalism in the AI age: towards an innovation process for responsible AI applications. In: Paper Presented at the the Joint Computation + Journalism European Data & Computational Journalism Conference 2023. 22-24 June 2023, Zurich, Switzerland.

[bibr50-14648849251317150] Prenger M Deuze M (2017) A history of innovation and entrepreneurialism in journalism In Remaking the News: Essays on the Future of Journalism Scholarship in the Digital Age. Pablo J. Boczkowski, C. W. Anderson. DOI: 10.7551/mitpress/10648.003.0022.

[bibr51-14648849251317150] Reporters Sans Frontières (RSF) (2023) Paris charter on AI and journalism. Retrieved from. https://rsf.org/sites/default/files/medias/file/2023/11/ParischarteronAIinJournalism.pdf.

[bibr52-14648849251317150] Roe J Perkins M (2023) ‘What they’re not telling you about ChatGPT’: exploring the discourse of AI in UK news media headlines. Humanities and Social Sciences Communications 10: 753. DOI: 10.1057/s41599-023-02282-w.

[bibr53-14648849251317150] Royal Charter for the Continuance of the British Broadcasting Corporation, Cm. 9365 (2016). London: HM Stationery Office. https://downloads.bbc.co.uk/bbctrust/assets/files/pdf/about/how_we_govern/2016/charter.pdf.

[bibr54-14648849251317150] Schmitz Weiss A Domingo D (2010) Innovation processes in online newsrooms as actor-networks and communities of practice. New Media & Society 12(7): 1156–1171. DOI: 10.1177/1461444809360400.

[bibr65-14648849251317150] Simkute A Luger E Jones B , et al. (2021) Explainability for experts: A design framework for making algorithms supporting expert decisions more explainable. Journal of Responsible Technology. doi: 10.1016/j.jrt.2021.100017.

[bibr55-14648849251317150] Simon FM (2023) Escape me if you can: how AI reshapes news organisations’ dependency on platform companies. Digital Journalism. DOI: 10.1080/21670811.2023.2287464.

[bibr56-14648849251317150] Sloane M Moss E Awomolo O , et al. (2022) Participation Is Not a Design Fix for Machine Learning. EAAMO ‘22. New York, NY: Association for Computing Machinery, 1–6. DOI: 10.1145/3551624.3555285.

[bibr57-14648849251317150] Vinsel L Russell AT (2020) The Innovation Delusion: How Our Obsession with the New Has Disrupted the Work that Matters Most. New York: Currency.

[bibr58-14648849251317150] Wagemans A Witschge T (2019) Examining innovation as process: action research in journalism studies. Convergence 25(2): 209–224. DOI: 10.1177/1354856519834880.

[bibr59-14648849251317150] Witschge T Deuze M (2020) From suspicion to wonder in journalism and communication research. Journalism & mass communication quarterly 97(2): 360–375.

