# Supplementary material for: Action research at the BBC: Interrogating artificial intelligence with journalists to generate actionable insights for the newsroom
Source: Journalism (Lond). 2025 Jan 29;26(8):1708–25. doi: 10.1177/14648849251317150 (PMC12244492; doi:10.1177/14648849251317150)
Supplement: Supplemental Material - Action research at the BBC: Interrogating artificial intelligence with journalists to generate actionable insights for the newsroom [file sj-pdf-1-jou-10.1177_14648849251317150.pdf]

Futures Thinking with Journalists

Resource Pack

Why futures thinking?

Futures thinking is an umbrella term for approaches to gathering insights and intelligence about possible futures. It can form an important part of responsible research and innovation (RRI) processes by helping people and their organisations anticipate impacts and implications of sociotechnical development, identify and mitigate risks, and open up discussion about desirable futures. It can also be a way to bring different parts of an organisation together to share knowledge, learn from each other's personal experience and professional expertise, and work productively together.

Why journalism?

Journalism today relies on an increasing array of technologies, which are changing at pace. News organisations and journalists must be prepared not only to react to emerging technologies but to anticipate and plan for their editorial, ethical, and practical implications – and understand how these technologies might be shaped to align with the values of responsible journalism. Making this a priority can help to build resilient, responsible, and sustainable newsrooms of the future that serve the public interest. For this work, researchers, designers, and newsroom innovators need new tools and methods – including futures thinking.

A speculative design resource pack

This resource pack is inspired by an approach called speculative design, in which provocative artefacts and scenarios are created to prompt debate and deliberation about potential future technologies and stimulate reflection and critique about their implications. It includes a set of example design provocations and guidance about how to use them, alongside a corresponding set of templates that can be tailored to your needs and tips on how to design and deploy your own provocations.

THE UNIVERSITY of EDINBURGH BBC R&D PETRAS PRETTY GOOD STUDIO

Authors: Dr Bronwyn Jones, University of Edinburgh & BBC, Dr Rhianne Jones, BBC R&D, Prof Ewa Luger, University of Edinburgh. Design: Pretty Good Studio

How to use this pack

This is an interactive document designed to allow participants to write their ideas, comments, and reactions directly into text boxes. Alternatively, it can be printed out and filled in manually. It is intended to be used as part of a workshop or discussion session to provide provocations as focus points for discussion and generate a written record of participants contributions, but it could also be distributed as a standalone exercise book alongside bespoke guidance.

We suggest you:

- Read the methodological guidance and tips
- Look at the examples of pre-designed speculative artefacts (related to generative AI) – or use them if this is a topic you want to explore
- Then see if the suggested templates would be useful for your purposes - you could fill them in ready to deploy with workshop participants (pre-design), or work with journalists to decide what to include (co-design)

What’s included

CLICK PAGE NUMBER TO JUMP

Guidance & Tips > 05-07

What If...? > 08-21

What might the jobs of the future look like? 09

What might next generation production systems look like? 11

Storyboarding the future 16

Mapping your insights 20

Templates > 22-30

Guidance & Tips

What can you do with speculative design?

How you use speculative design for futures thinking will depend on what you want to find out, what resources you have, and what context you're working in – but these are the two main processes you might want to consider:

- **Co-designing** speculative artefacts with journalists
- **Deploying pre-designed** speculative artefacts with journalists

Co-designing involves an iterative and interactive process of working with journalists to interrogate (a selection of) technological capabilities and affordances that are emerging or may emerge in the future, connecting them to the journalistic field (e.g., workflows, practices, processes, infrastructures etc.), and then translating these projections of future possibilities into tangible artefacts. These speculative artefacts (i.e. things - technology, document, object etc. - that could exist in the future) can then be deployed as provocations with other journalists or stakeholders to explore their reactions, thoughts, hopes and fears – or to probe further what impacts, implications, risks and opportunities they might raise. This approach has the advantage of leveraging the expertise of stakeholders who have expertise and investment in the topic being investigated but is resource, skill, and time intensive.

Deploying pre-designed speculative artefacts without the involvement of journalists/stakeholders is another option, which can be less resource-intensive and quicker to achieve. In this case, you/your team would draw from literature and other resources to create the artefacts before taking them to journalists for discussion and deliberation. However, this can risk missing the important insights of experts in the field and excluding the voices of those likely to be impacted by the changes under investigation.

Check out our examples

In the following section you'll find a selection of speculative artefacts related to a set of technologies called Generative AI. They were co-produced with journalists in workshops by looking at current trends and innovations in media and communications technology, alongside existing editorial workflows and practices. Our aim when designing these provocations was to consider not just what might be possible in the future, but also what might be plausible applications of generative AI.

We then used them as provocations for discussion with journalists and other stakeholders as an exercise to identify what questions and risks generative AI might pose for journalism, which we further developed into an infographic and shared with other editorial workers as a resource for learning.

What If...?

Exercises with Speculative Design

What might the jobs of the future look like?

When technologies in the workplace change, so can the nature of everyday work, job opportunities, and labour relations. This artefact - a job description - imagines the role of a Prompt Engineer in the newsroom, who takes on a role working with generative AI tools. It prompts us to ask:

- What skills could be needed if generative AI is incorporated into news production, and why?
- How could this impact the workforce and existing practices?
- Who has control or involvement in making employment decisions? - and what might they want or think?
- What could this mean for journalistic standards?
- What is desirable/undesirable about this situation and why?
- How could the issues it raises be mitigated and who would be responsible?

Job Description

Careers

Position:

Prompt Engineer/Journalist

NEXT JOB

JOB DESCRIPTION:

Fancy being at the forefront of applying artificial intelligence (AI) to improve news coverage? Want to be part of a multi-award winning AI-human collaborative team?

We're looking for a new age journalist with skills in prompting generative pretrained transformers like ChatGPT and experience producing images, videos, and graphical storyboards with tools from our partners (like DALL-E, Midjourney, Synthesia).

We need a talented prompt engineer with journalistic skills to leverage their experience crafting generative AI outputs and develop new skills covering our busy news patch. You will deliver the highest quality AI-generated content for online, TV and radio, as well as for social platforms.

The successful candidate will combine their track record of creative thinking with new skills for creative prompting and AI-augmented reporting – not to mention their thorough grasp of Law (including new AI regulations) and Editorial Guidelines (including recent generative AI principles).

ROLE RESPONSIBILITY:

Prompt engineering to elicit the best generative AI content possible

Co-writing and co-editing with AI to produce broadcast-quality scripts for radio and TV programmes and bulletins

Providing your voice and face to train our synthetic avatar (*see terms and conditions)

Scoping out and incorporating the latest AI technology

Upskilling your colleagues in emerging skills

Working to tight deadlines

Confidence in dealing with complex technology-editorial and legal issues

*We will own the rights to this virtual avatar but you will receive royalties relational to its use in perpetuity. Please discuss at offer stage.

SALARY:

In recognition of the high demand for this combination of skills and to benchmark against the wider technology industry rates, this role will be matched to an Editor-level salary. Further discussion at interview stage.

Excellent career progression – opportunity to develop the job as abilities of AI progress and to work with our technology and research teams to improve our AI offering

Training and development – access to a wide range of state-of-the-art courses and certification (e.g. Grade 3 in Prompt Engineering, Level 4 Machine Co-operation)

Benefits – For every time saving through your skills at automating tasks, we offer a bonus day of leave.

Apply

Jump to the template to create your own

What might next generation production systems look like?

The tools and systems journalists work with every day change over time to incorporate new functionality, which influences the capabilities and agency of journalists as well as their workflows and processes. The following artefacts are all based on the interface of a content management system (CMS). They have a familiar layout but imagine how forms of artificial intelligence and automated text, image, and audio generation could be integrated into news production technologies. They prompt us to ask:

- Which journalistic tasks could generative AI play a role in and how?
- What are the capabilities and limitations of these AI systems?
- How might deployment in a CMS change the nature of news production in positive and negative ways?
- What benefits and risks might this pose? To who/what?
- What are the legal and professional implications of integrating AI in these ways?
- What would need to be put in place for these types of AI applications to be safe, secure, ethical, and operating within journalistic values and standards?
- What do journalists actually want emerging AI technologies to do - and what should be kept within the purview of editorial workers?
- How could audiences/news users be impacted?

CMS1

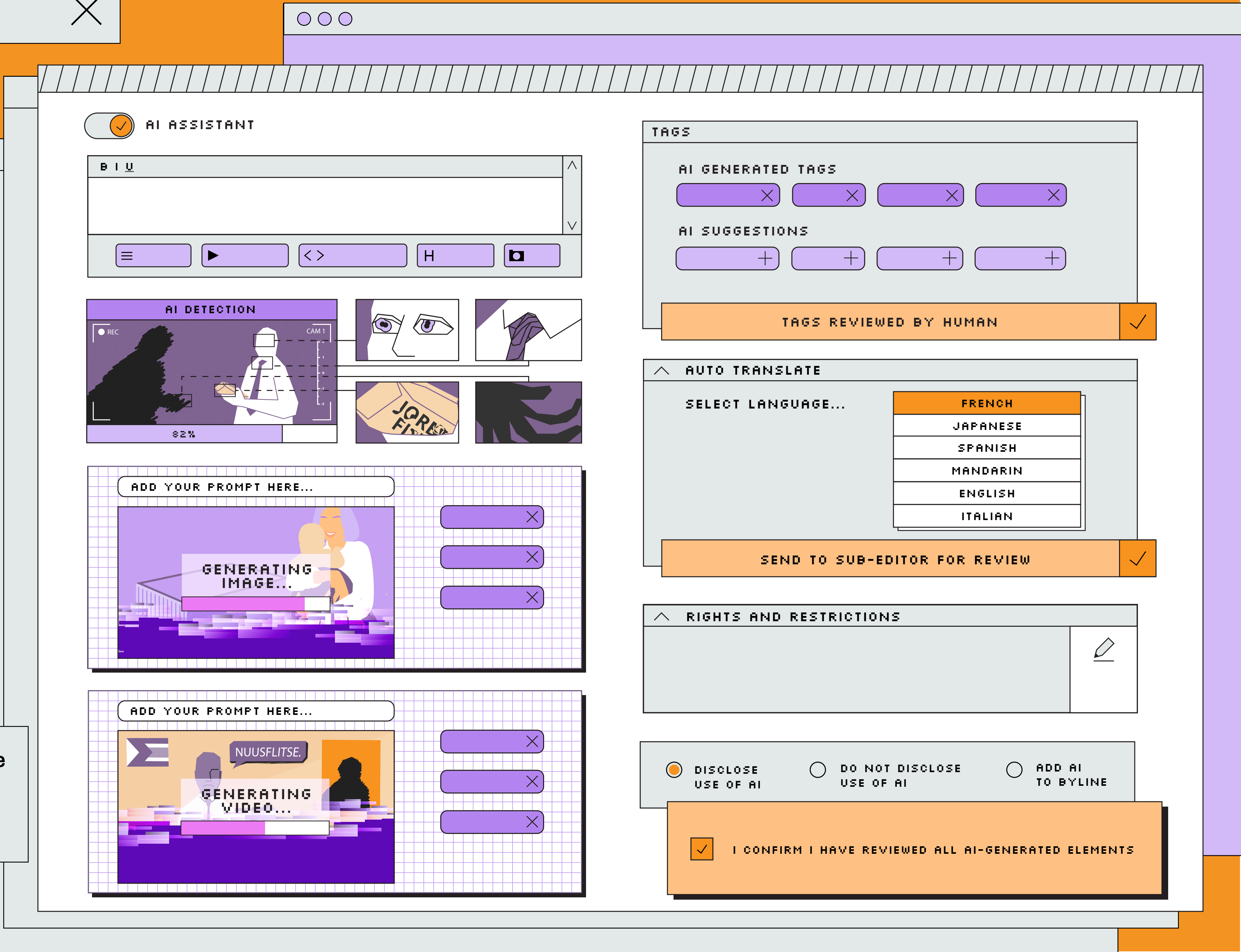

Jump to the template to create your own

CMS2

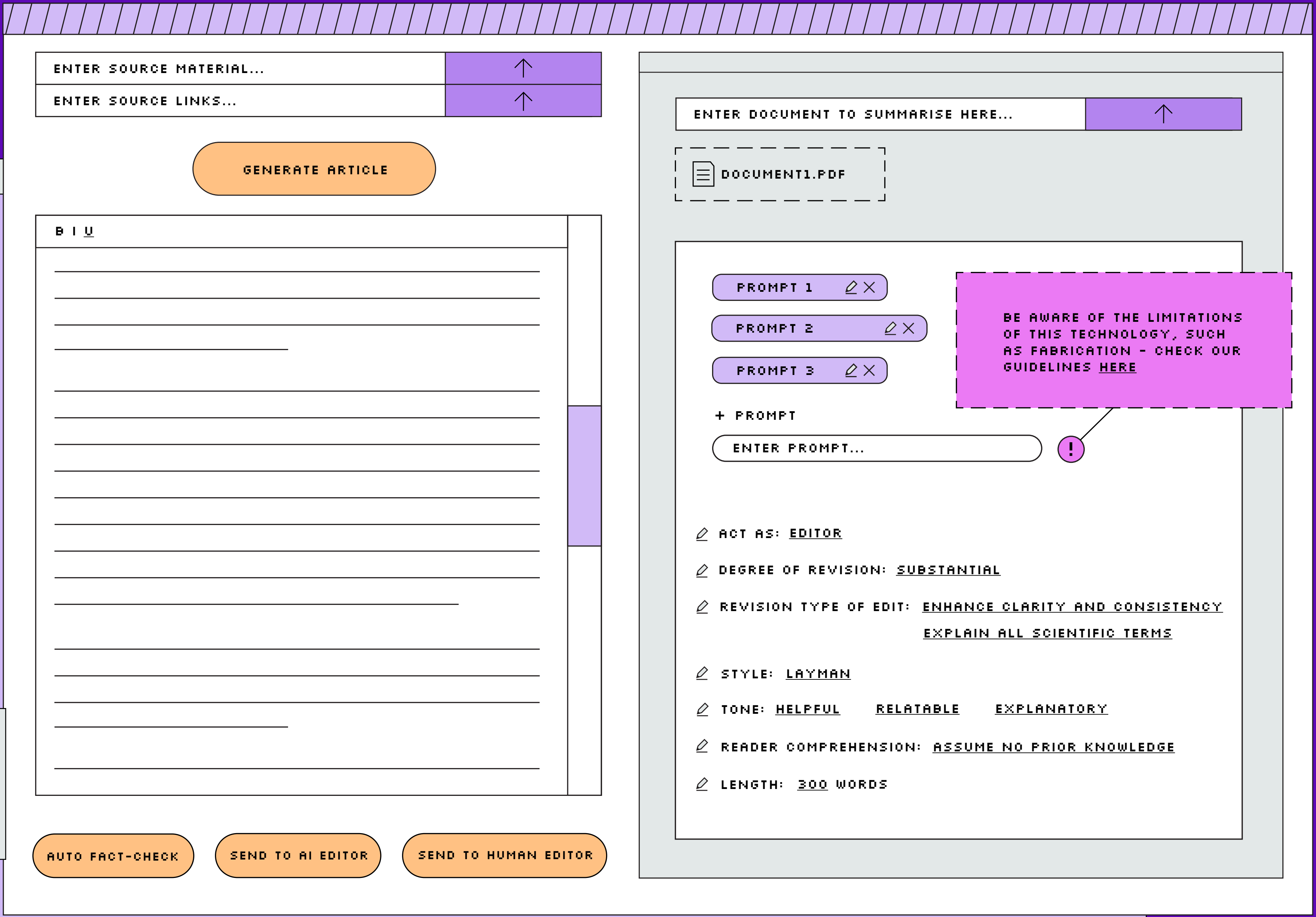

Jump to the template to create your own

CMS3 X

GENERATE...

IMAGE VIDEO STORYBOARD

ENTER STORY URL...

BBC.CO.UK/NEWS/850272 X

EXTRACT KEY STORY POINTS

SEE TIPS ON PROMPT ENGINEERING FROM OUR GUIDE

ENTER PROMPTS...

OFFICE ENVIRONMENT X
COLLABORATION X
TEAM PROJECT X

ENTER PROMPTS...

UPBEAT SUMMER PIANO X

GENERATE VIDEO

GENERATE MUSIC

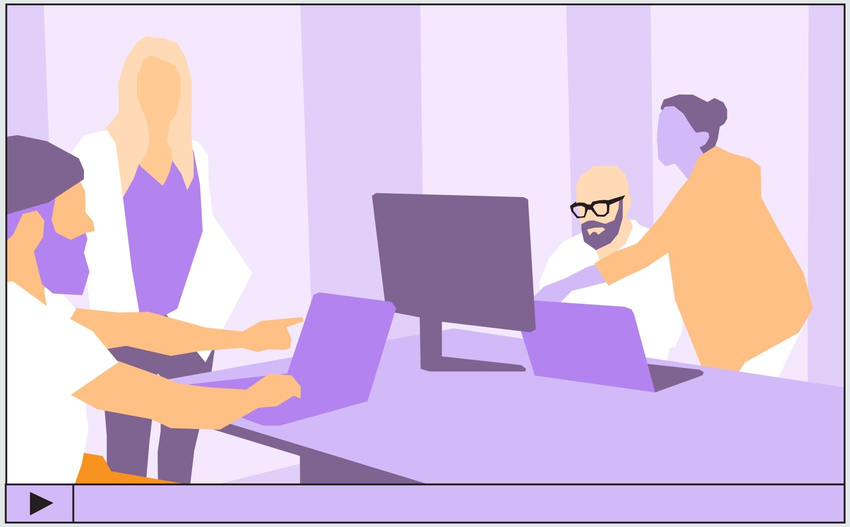

I CONFIRM I HAVE CHECKED THIS VIDEO FOR ERRORS AND PROBLEMATIC MATERIAL.

SCAN FOR COPYRIGHT INFRINGEMENT

ADD AI WATERMARK

Jump to the template to create your own

CMS3

GENERATE...

IMAGE VIDEO STORYBOARD

NOT TO BE USED FOR SENSITIVE STORIES. SEE GUIDANCE

ENTER STORY URL...

!

EXTRACT KEY STORY POINTS

- WAITING TIMES FOR DENTAL APPOINTMENTS HAVE RISEN BY 50% IN A YEAR.
- MORE PEOPLE ARE TURNING UP TO A&E AND THEIR GP SURGERY WITH PAINFUL INFECTIONS.
- THE GOVERNMENT SAYS IT HAS ALLOCATED £50M TO TRAIN MORE DENTISTS.

CHOOSE STYLE...

GENERATE STORYBOARD

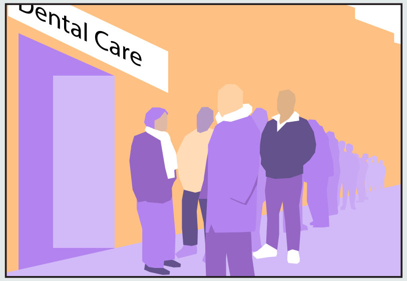

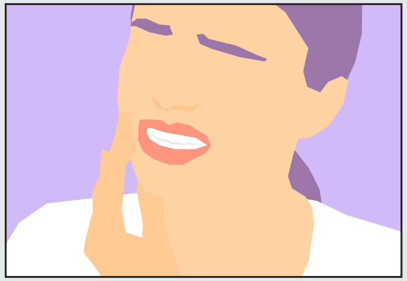

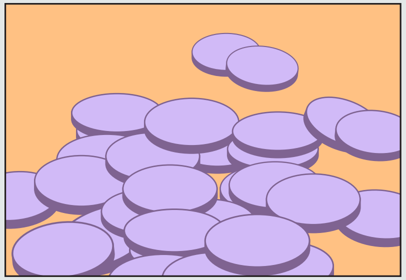

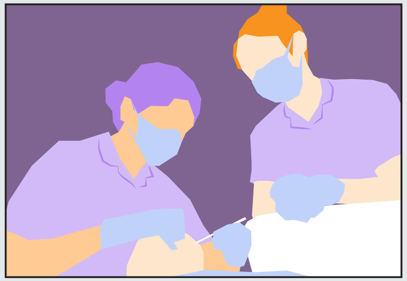

SEND TO EDITOR REGENERATE

Jump to the template to create your own

Storyboarding the future

Storyboards can be powerful ways to explore how scenarios involving technologies might play out in different contexts. The following examples depict two scenarios: one tells the story of a problematic/negative outcome of AI-generated synthetic media and the other depicts a useful/positive outcome.

Storyboard 1: Deepfakes being used to disrupt of an election

Storyboard 2: Auto-translation of audio, text, and video into multiple languages

Journalists/participants can fill in the text boxes to describe what they think is happening and if in a group, discuss the issues with each other. Alternatively, they can draw their own scenarios. This activity can help stakeholders anticipate potential opportunities or benefits as well as challenges, risks, and implications linked to a particular application/use of technology.

These storyboards prompt us to ask:

- What possible sequence of events might occur if synthetic media was used by bad actors to disrupt news reporting?
- What can journalists and news organisations do to protect themselves? To ensure their reporting remains accurate and trustworthy?
- How could this impact audiences? And what would be needed to mitigate against the worst impacts?
- What might be the most beneficial applications of synthetic media for news reporting? Who could this help and how?
- What would be necessary to make this potential future a reality?

Storyboard 1

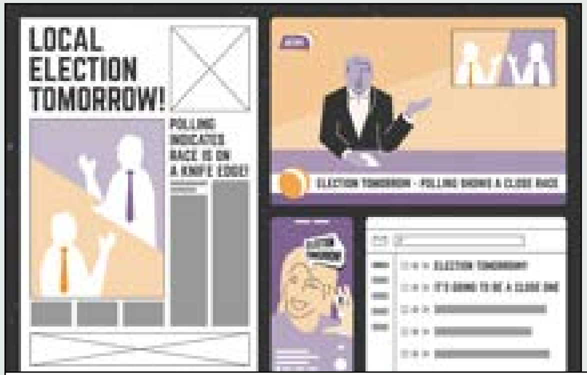

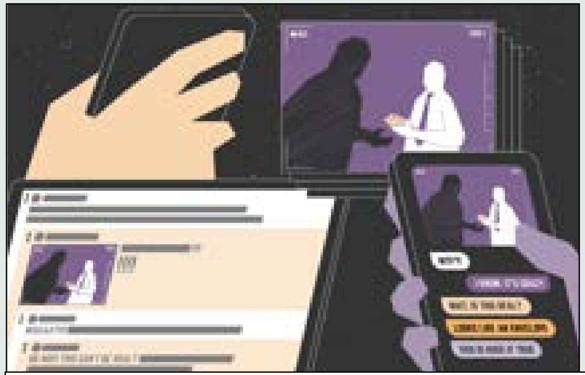

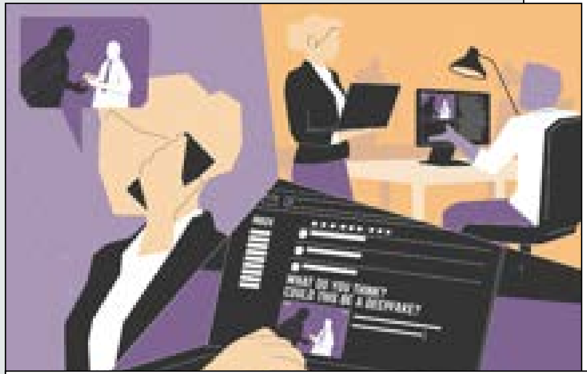

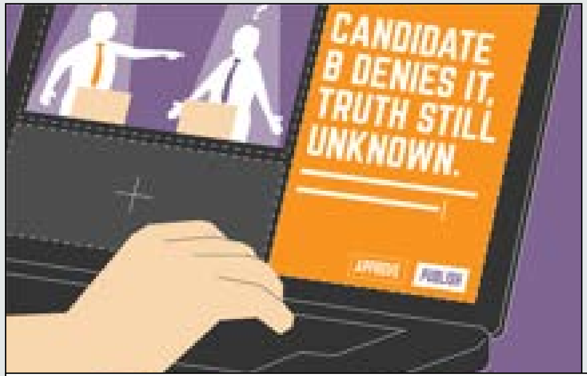

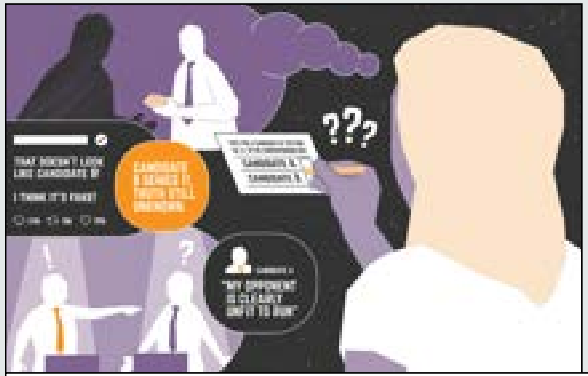

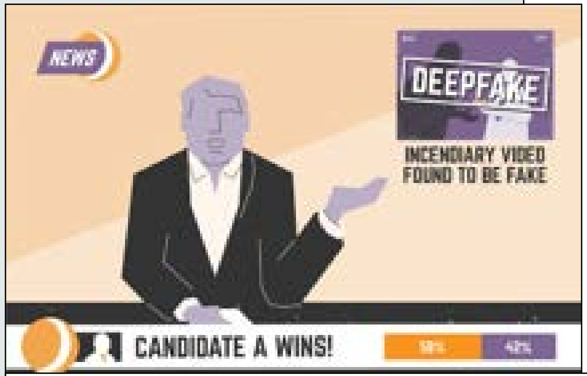

Storyboard 2

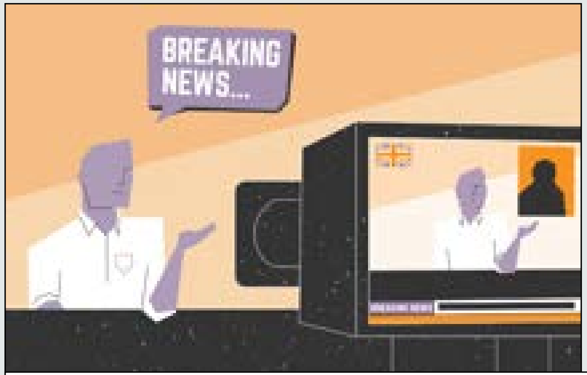

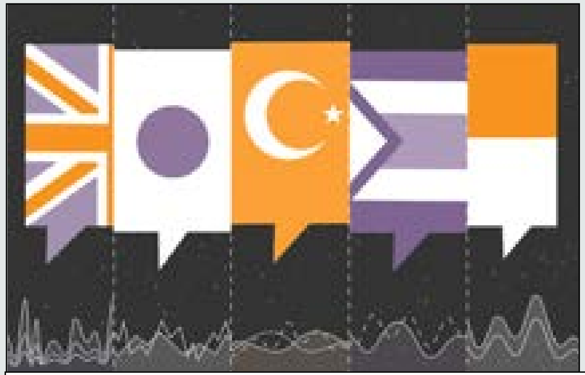

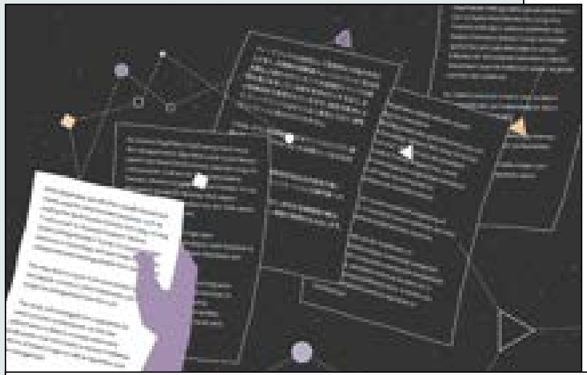

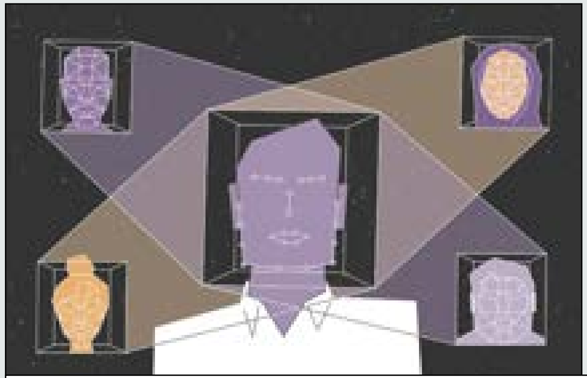

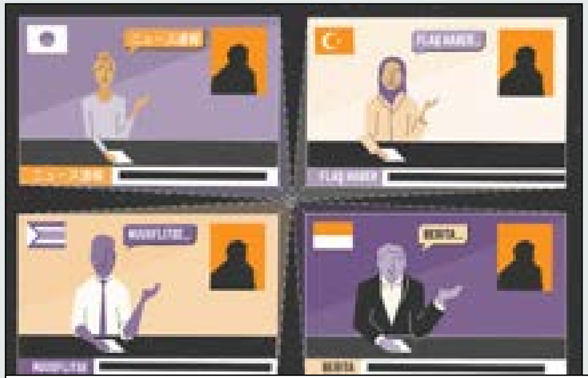

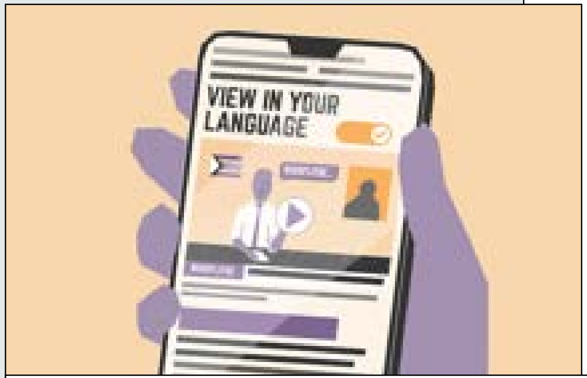

Storyboard 2

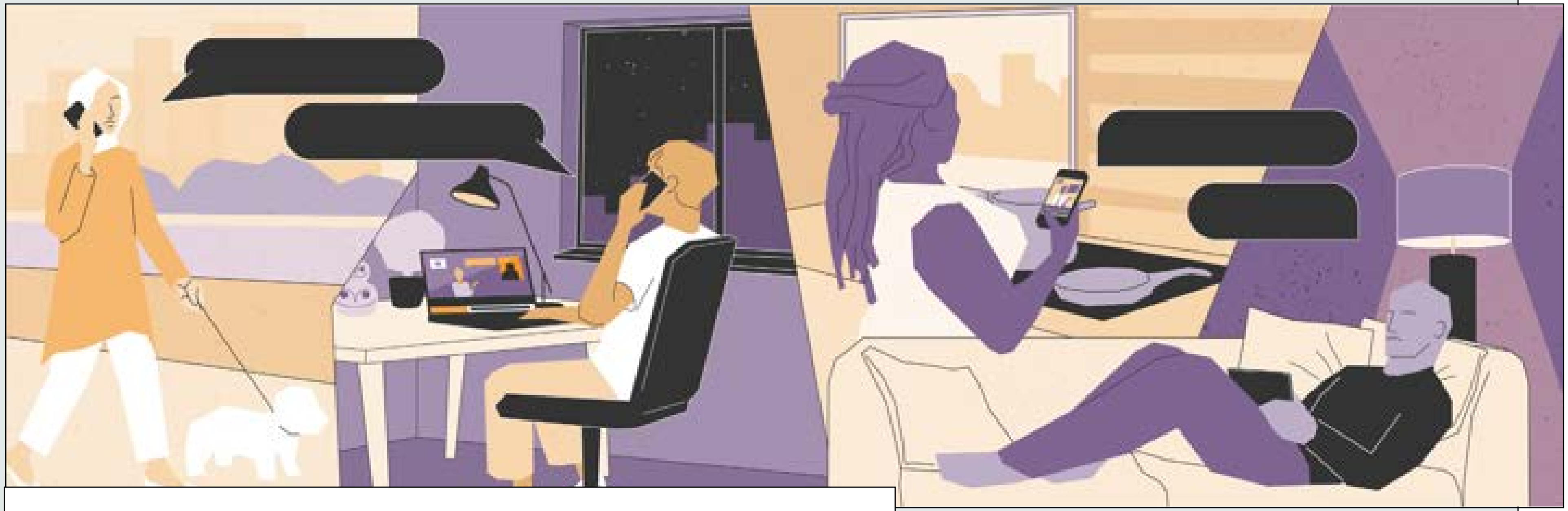

Jump to the template
to create your own

Mapping your insights

Co-designing and deploying speculative artefacts is a way to generate insights about a topic or technology - but capturing these in the moment can be hard. We recommend closing a workshop or discussion by mapping out the key questions/challenges/opportunities/risks - whatever angle you want to focus on.

We asked journalists to think about what they'd learnt and discussed in relation to the speculative artefacts and generate an infographic answering: **What key questions does generative AI raise for journalism?**

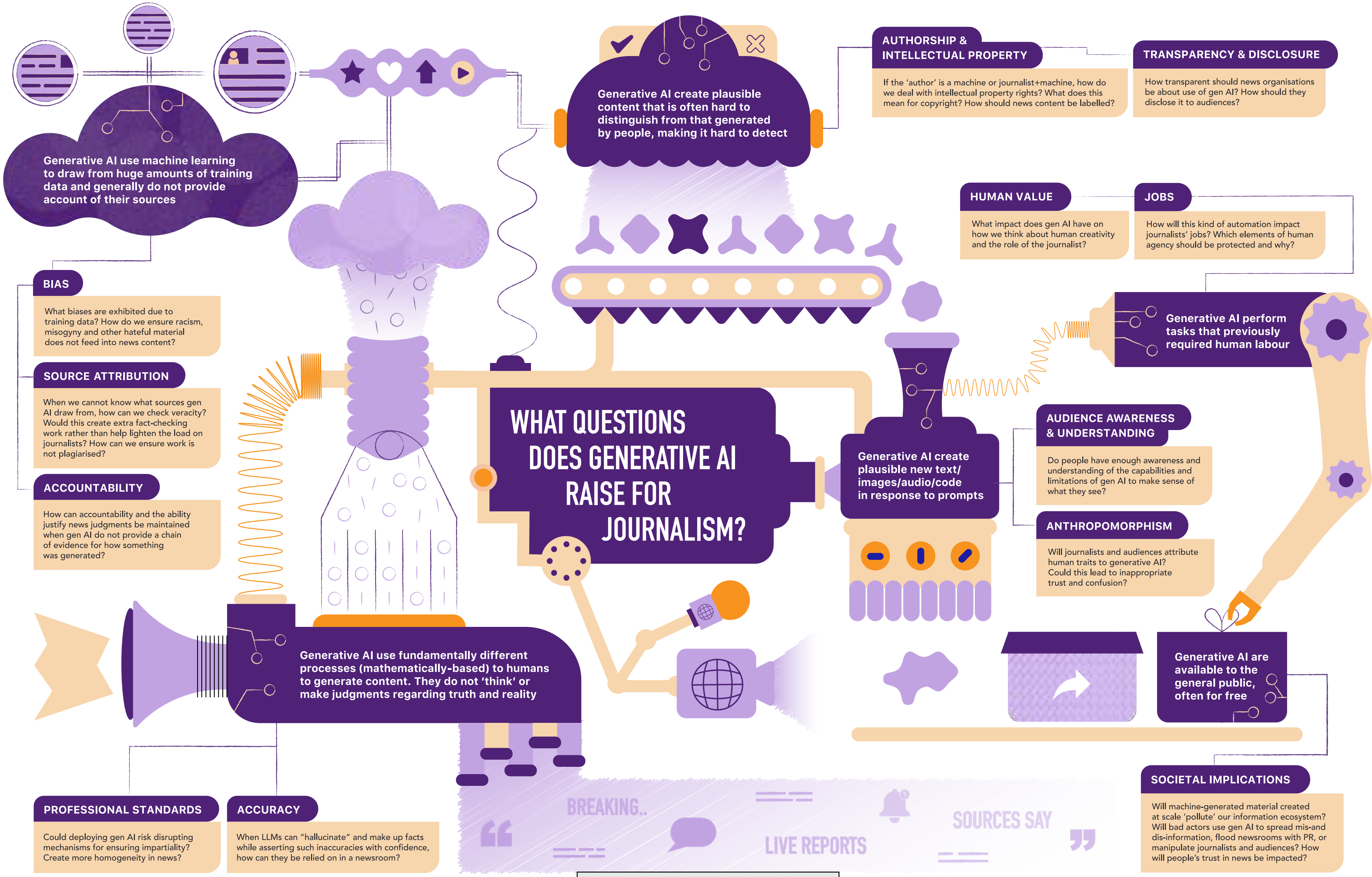

Jump to the template to create your own

Templates

Design Your Own Speculative Artefacts

What skills and responsibilities might be needed in the future of journalism?

Careers

Position:

NEXT JOB

JOB DESCRIPTION:

ROLE RESPONSIBILITY:

SALARY:

Apply

What might the next generation CMS be able to do?
What capabilities could it have?

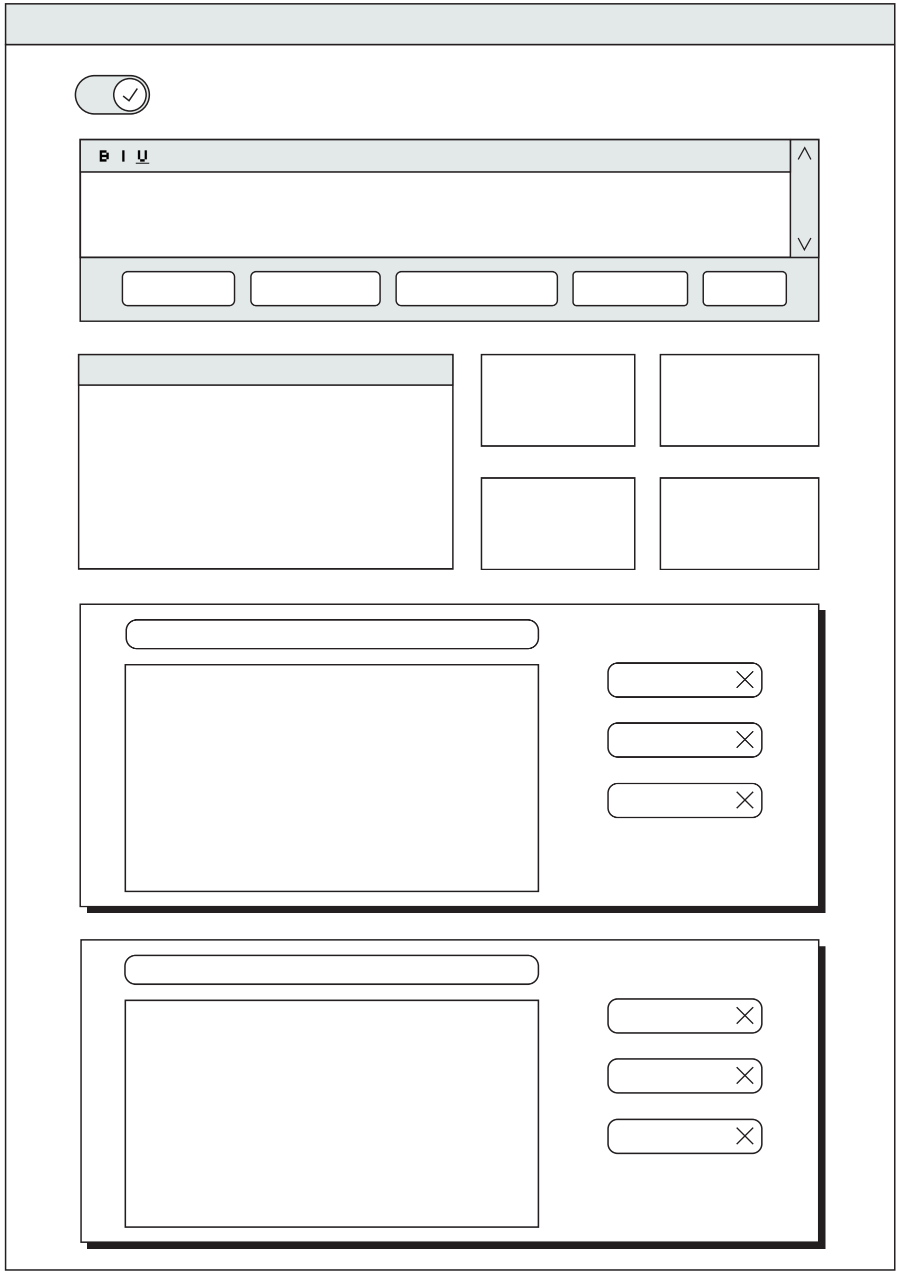

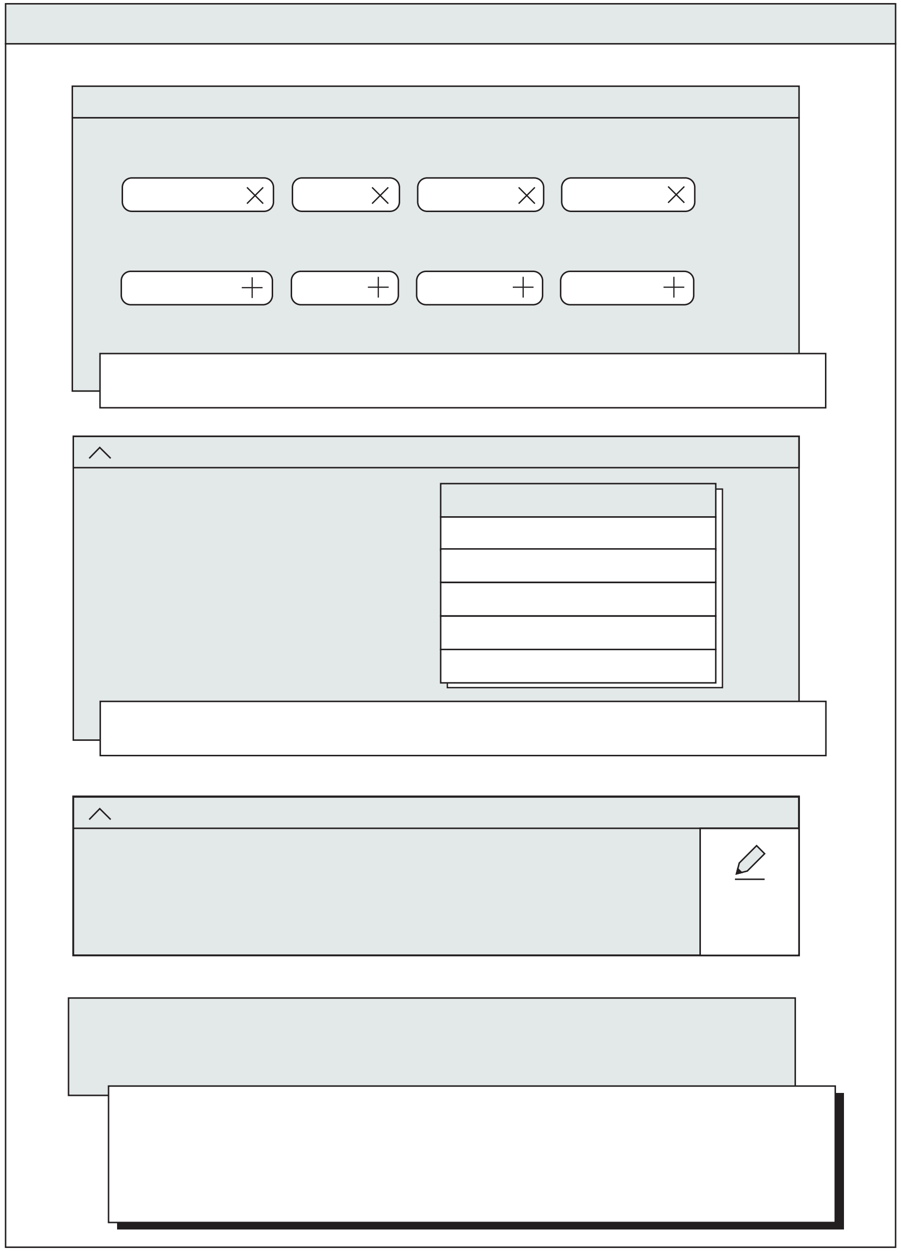

How might a CMS in future be involved in writing journalistic prose?
What issues could this raise?

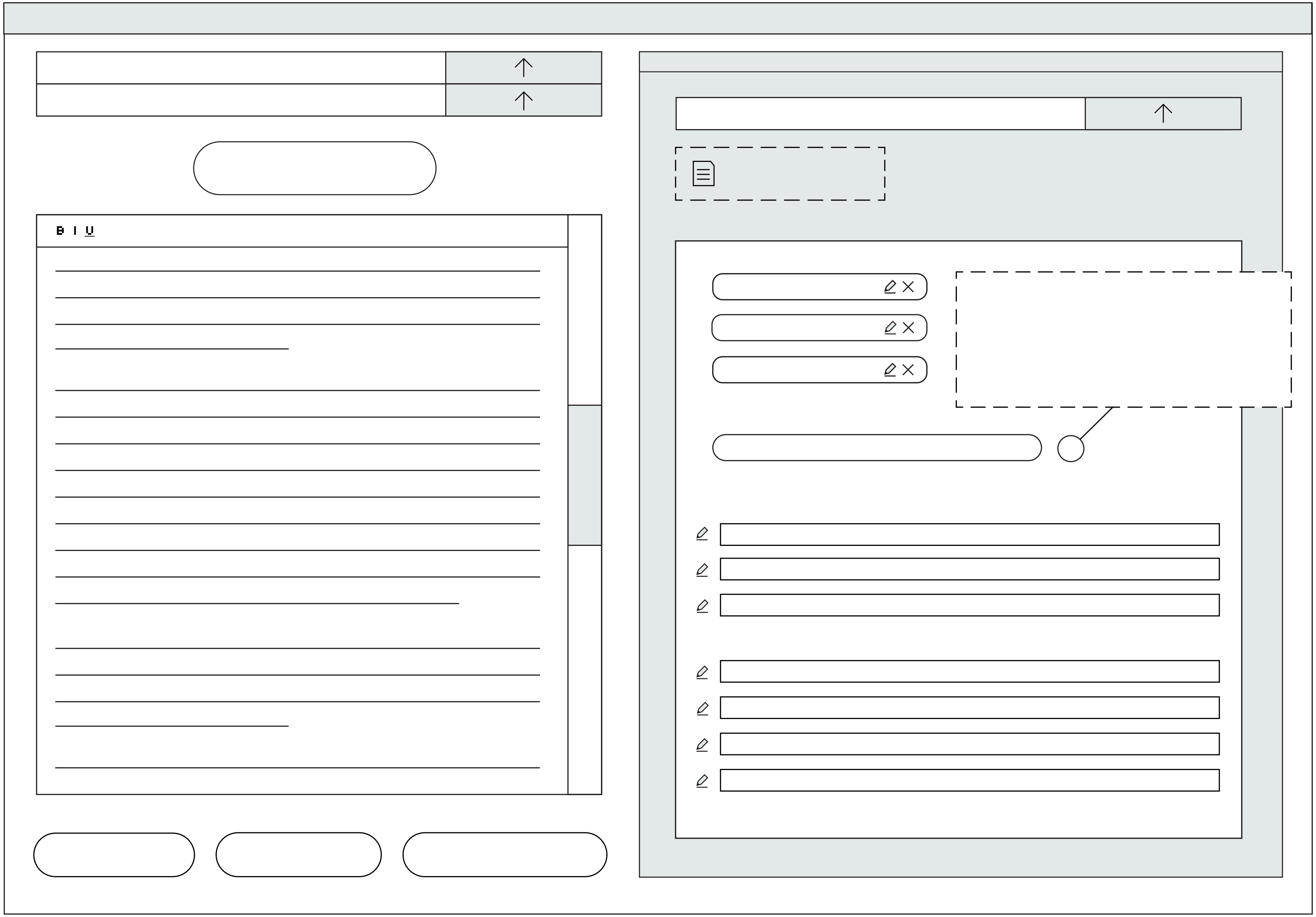

What capabilities for dealing with video might a next generation CMS have?
And what legal or professional questions could this pose?

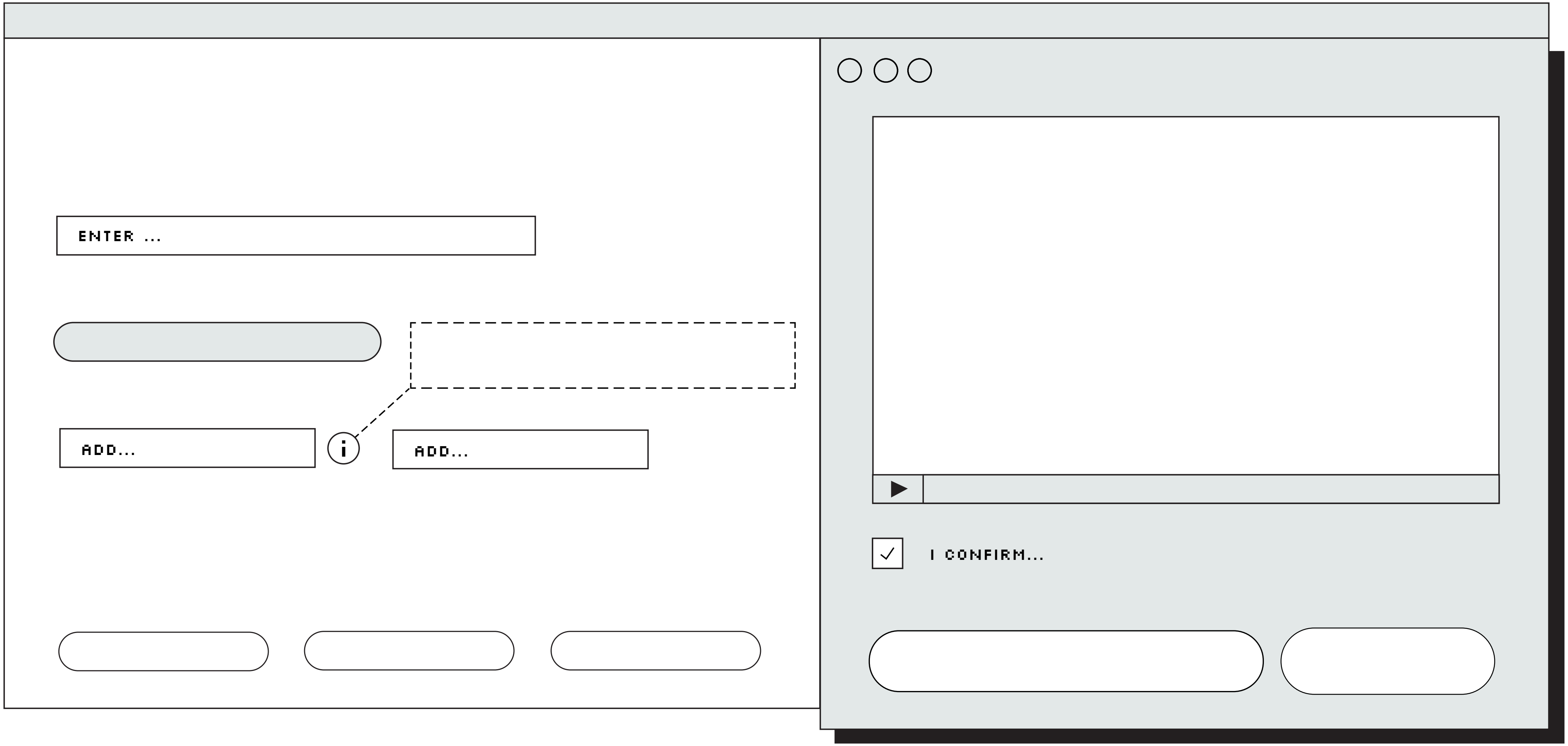

What would journalists want from a CMS with new functionality and why?

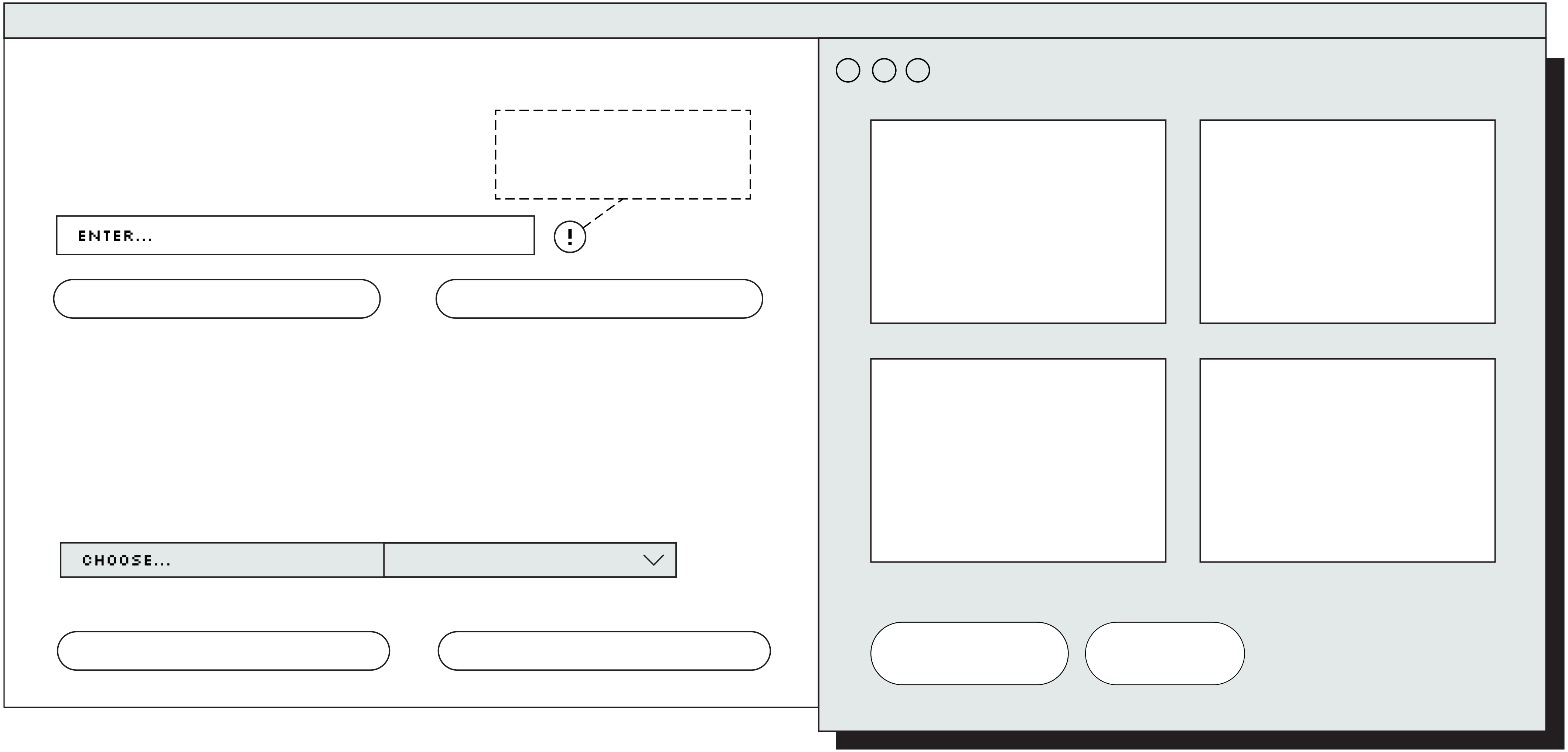

You could try other formats, for example:
What might news coverage of this technology look like?

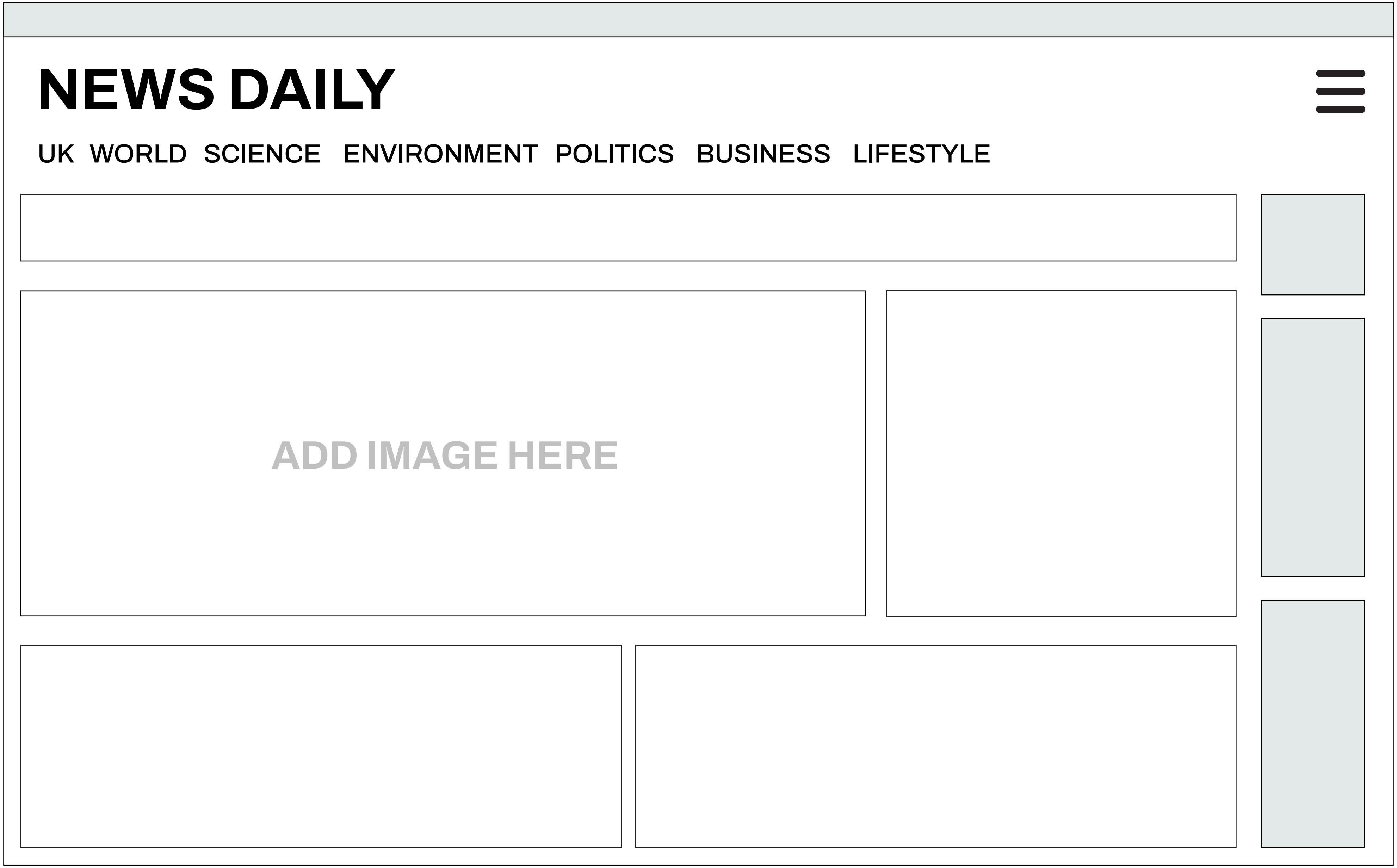

Create your own storyboard

What questions/opportunities/challenges/risks does the topic you're discussing pose?
Can you map the key issues out?

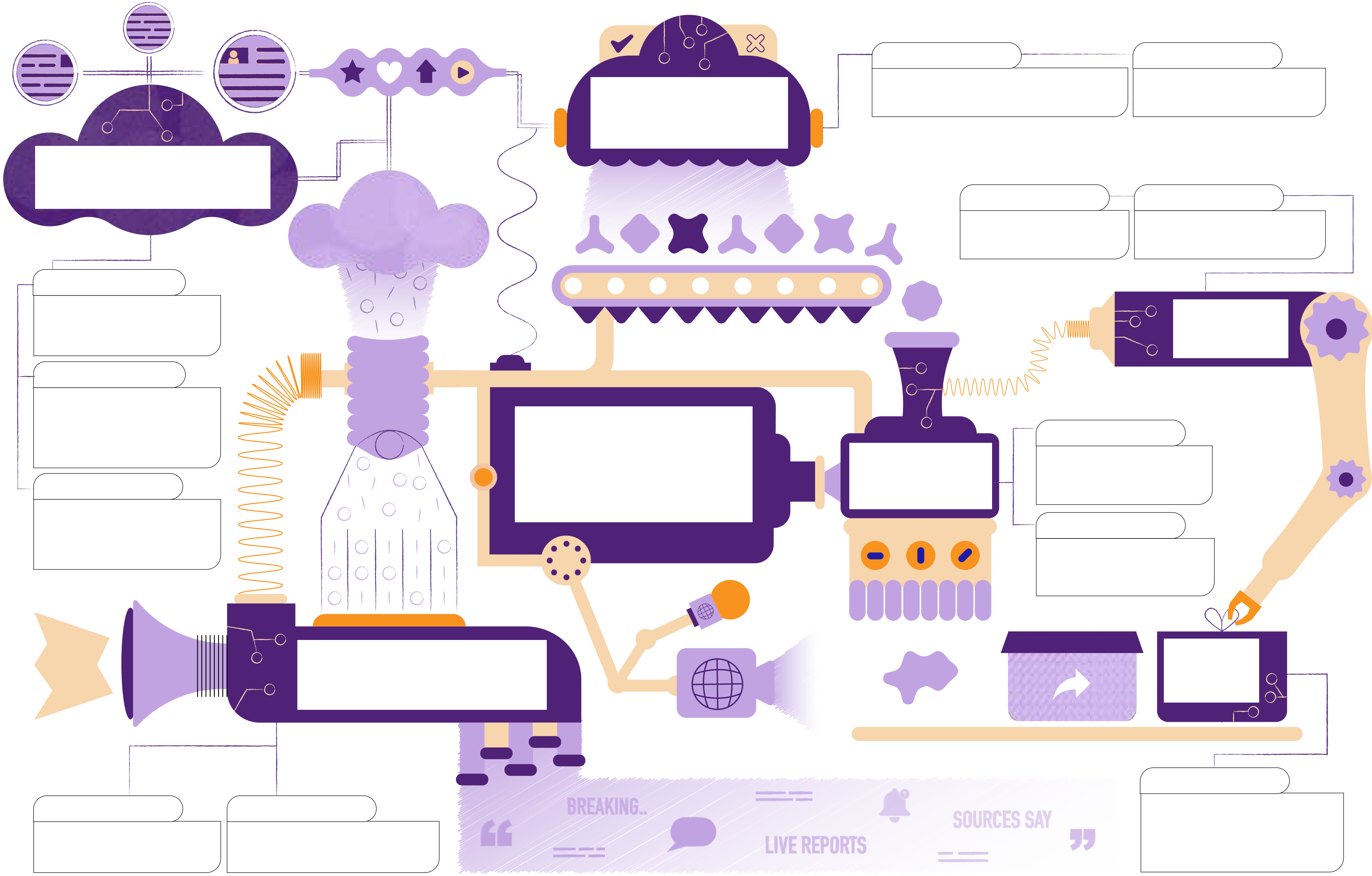

Designing desirable news futures

We hope this pack helps you think about and plan for a future that prioritises responsible development and use of technology in journalism!

Please share this pack with your colleagues and if you have any questions, contact: Bronwyn.jones@bbc.co.uk or Rhia.jones@bbc.co.uk

The Futures Thinking with Journalists Resource Pack was produced as part of the BBC's Responsible Innovation work stream in a collaboration with the University of Edinburgh and PETRAS National Centre of Excellence.

Authors: Dr Bronwyn Jones, University of Edinburgh & BBC, Dr Rhianne Jones, BBC R&D, Prof Ewa Luger, University of Edinburgh. Design: Pretty Good Studio
